# Systemic acquired resistance signaling molecule N-hydroxypipecolic acid is involved in Age-related resistance in *Arabidopsis thaliana*

**DOI:** 10.1093/plphys/kiag302

**Published:** 2026-05-23

**Authors:** Garrett Nunn, Jacob Lund, Natalie Belu, Wantao N Xiao, Rowan Brookman, G Brian Golding, Robin K Cameron

**Affiliations:** Department of Biology, McMaster University, Hamilton, Ontario, Canada; Department of Biology, McMaster University, Hamilton, Ontario, Canada; Department of Biology, McMaster University, Hamilton, Ontario, Canada; Department of Biology, McMaster University, Hamilton, Ontario, Canada; Department of Biology, McMaster University, Hamilton, Ontario, Canada; Department of Biology, McMaster University, Hamilton, Ontario, Canada; Department of Biology, McMaster University, Hamilton, Ontario, Canada

## Abstract

Little is known about how mature *Arabidopsis thaliana* (Arabidopsis) plants become competent to initiate age-related resistance to normally virulent *Pseudomonas syringae* pv. *tomato* (*Pst*). RNA sequencing was used to identify genes that contribute to age-related resistance by comparing the response to *Pst* in young, susceptible plants 3.5 weeks postgermination with mature Age-related resistance–competent plants 6 weeks postgermination. Comparative transcriptomics revealed that age-related resistance shares many genes with pathogen-associated molecular pattern–triggered immunity, effector-triggered immunity, and systemic acquired resistance, providing additional evidence for the emerging idea that plant immune pathways are intertwined and interconnected. Age-related resistance–responding leaves expressed genes for the biosynthesis of N-hydroxypipecolic acid, a signaling molecule involved in systemic acquired resistance during the establishment of a primed state in systemic leaves. N-hydroxypipecolic acid biosynthesis mutants (*ald1-1*, *fmo1-1*) had age-related resistance defects and accumulated less intercellular salicylic acid than wild-type Col-0, indicating a requirement for functional N-hydroxypipecolic acid biosynthesis genes for intercellular salicylic acid accumulation during age-related resistance. Salicylic acid perception by NPR1/4 proteins was required for age-related resistance, as demonstrated by the age-related resistance–defective phenotype of the salicylic acid–signaling mutant, *npr1-1 npr4-4D*. N-hydroxypipecolic acid biosynthesis genes were still expressed in *npr1-1 npr4-4D*, suggesting that, similar to systemic acquired resistance, N-hydroxypipecolic acid functions upstream of NPR1-mediated signaling during age-related resistance. Unlike age-related resistance–incompetent plants (3, 4, 5 weeks postgermination untreated), age-related resistance–competent plants (6 weeks postgermination untreated) expressed N-hydroxypipecolic acid biosynthesis genes, accumulated N-hydroxypipecolic acid, and expressed immunity-associated cell surface receptors *RLP23* and *RLP28*, supporting the idea that N-hydroxypipecolic acid contributes to initiating a developmentally controlled age-related resistance–competent primed state in mature Arabidopsis that is similar to the primed state established in distant leaves during systemic acquired resistance.

## Teaser

Mature healthy plants accumulate N-hydroxypipecolic acid, suggesting Age-related resistance includes developmentally controlled priming similar to pathogen-induced priming in Systemic acquired resistance.

## Introduction

Many plant species develop resistance to normally virulent pathogens as they age, a phenomenon known as age-related resistance (ARR) (Whalen 2005; Develey-Rivière and Galiana 2007; Carella et al. 2015). ARR has been observed in the model plant species *Arabidopsis thaliana* in response to biotic stressors, including several insect species (Mao et al. 2017), the biotrophic oomycete *Hyaloperonospora parasitica* (Rusterucci et al. 2005), and the hemibiotrophic bacterial pathogen *Pseudomonas syringae* (Kus et al. 2002). Young [3–4 weeks postgermination (wpg)] *Arabidopsis* plants grown in short 9-h days were susceptible to virulent strains of *P. syringae* pv. *tomato* (*Pst*), while mature plants (6–7 wpg) grown in similar conditions displayed ARR, as demonstrated by 10- to 100-fold reductions in bacterial levels compared with young plants (Rusterucci et al. 2005). The ARR response in *Arabidopsis* is associated with the transition to flowering, which led to the idea that a developmentally regulated receptor becomes activated at the transition to flowering, resulting in ARR competence (Rusterucci et al. 2005). However, in subsequent studies, young plants induced to flower early by transient exposure to long days did not display ARR, and analysis of early- and late-flowering mutants demonstrated that the transition to flowering is not sufficient or required for ARR competence (Wilson et al. 2013).

Compared with our knowledge of ARR in mature *Arabidopsis*, we know a great deal more about young plant resistance responses: pathogen-associated molecular pattern (PAMP)–triggered immunity (PTI) and effector-triggered immunity (ETI). A well-studied PTI response is initiated upon binding of the PAMP flg22 (a 22–amino acid epitope of bacterial flagellin) to the plant receptor-like kinase FLAGELLIN SENSITIVE2 (FLS2) (Chinchilla et al. 2007). Initiation of PTI results in a reactive oxygen species burst, transcriptional changes, accumulation of plant defense hormones, callose deposition, accumulation of phytoalexins, and pathogenesis-related proteins (PRs) (Hudson et al. 2024). These defense responses result in resistance to biotrophic pathogens, including *P. syringae* (Toruño et al. 2016). Successful bacterial pathogens like *Pst* use a type 3 secretion system (T3SS) to secrete effector proteins into plant cells to suppress PTI and manipulate the host environment to favor pathogen growth, resulting in plant susceptibility (Toruño et al. 2016; Xin et al. 2018). For example, PTI in young plants is suppressed early during infection with virulent *Pst*, as demonstrated by suppression of callose formation (Hauck et al. 2003) and salicylic acid (SA) accumulation (Carviel et al. 2014). PTI-associated gene expression was also suppressed early during infection with virulent *Pst* in a T3SS-dependent manner, as demonstrated in experiments in which wild-type *Pst* was compared to T3SS-deficient *Pst* mutants during infection of Arabidopsis (Lewis et al. 2015).

ETI allows plants to detect pathogen effector proteins; for example, the *Arabidopsis* protein RESISTANCE TO PSEUDOMONAS SYRINGAE PV MACULICOLA1 (RPM1) receptor detects the phosphorylation of the plant protein RPM1-INTERACTING PROTEIN4 (RIN4) (Liu et al. 2011) by the *P. syringae* pv. *maculicola* effector AvrRpm1 (Ritter and Dangl 1995; Redditt et al. 2019) to initiate ETI. ETI initiation includes many of the same components as PTI (Ngou et al. 2021; Yuan et al. 2021), resulting in a similar defense response (Ngou et al. 2021; Yuan et al. 2023), with a few differences, such as a sustained influx of intracellular Ca^2+^ and programmed cell death (Wang et al. 2024). ETI and PTI in *Arabidopsis* include the biosynthesis and accumulation of SA (reviewed in Maruri-López et al. 2019). SA is thought to act primarily as a signaling molecule to upregulate the expression of defense genes via the transcriptional activator NONEXPRESSOR OF PR GENES1 (NPR1), a master regulator of plant immune responses, including PTI and ETI (Chen et al. 2017; Liu et al. 2020). Redox changes following treatment with SA result in the oligomeric to monomeric NPR1 transition (Tada et al. 2008), followed by NPR1-SA binding (Wu et al. 2016; Ding et al. 2018) and translocation of NPR1 into the nucleus (Kinkema et al. 2000; Mou et al. 2003), where it binds to the nuclear-localized TGACG-BINDING FACTOR (TGA) transcription factors (Zhou et al. 2000; Fan and Dong 2002), resulting in the accumulation of antimicrobial compounds, PR proteins, local resistance (PTI and ETI), and systemic acquired resistance (SAR) (reviewed in Bigeard et al. 2015; Chen et al. 2017; Yildiz et al. 2021).

In *Arabidopsis*, SAR is initiated in distant systemic leaves following local inoculation with virulent or avirulent strains of *P. syringae* (Cameron et al. 1994; Mishina and Zeier 2007). Long-distance mobile signals are produced in local leaves and travel cell to cell and via the phloem to distant leaves (Vlot et al. 2021), resulting in molecular changes, including chromatin remodeling at defense gene promoters to produce an immune ready/primed state (Jaskiewicz et al. 2011; Baum et al. 2019) and modest accumulation of SA and N-hydroxypipecolic acid (NHP), 1- to 2-fold higher than in untreated Arabidopsis leaves (Návarová et al. 2012; Cai et al. 2021). The immune-ready/primed leaves initiate a rapid and strong immune response to subsequent infection with normally virulent pathogens (Dempsey and Klessig 2012; Vlot et al. 2021).

Like SAR, ARR is an SA-dependent pathway, as demonstrated by the ARR-defective phenotypes in SA-deficient mutants (Kus et al. 2002). However, ARR was observed in mutants of NPR1 (Kus et al. 2002) and WHIRLY1 (WHY1), suggesting that NPR1-independent signaling may also contribute to ARR (Carella et al. 2015). SA also accumulates in the intercellular space where many bacterial pathogens grow and multiply during ETI (Carviel et al. 2014), PTI (Xiao et al. 2024), and ARR (Wilson et al. 2017). In leaves of young susceptible *Arabidopsis*, little intercellular SA accumulates in response to virulent *Pst*, which forms many large biofilm-like aggregates (Xiao et al. 2024), perhaps because virulent *Pst* uses its effectors and the phytotoxin coronatine (Zheng et al. 2012) to suppress plant defenses like SA accumulation (Toruño et al. 2016). In contrast, intercellular SA accumulation during both ARR (Wilson et al. 2017) and PTI (Xiao et al. 2024) is associated with very few small *Pst* biofilm-like aggregates. SA is estimated to accumulate to concentrations of 50 to 100 µM in leaf intercellular spaces of PTI-responding (Xiao et al. 2024) and ARR-responding plants, and similar SA concentrations inhibit *Pst* biofilm formation in vitro (Wilson et al. 2017). These studies support the idea that SA acts as an antibiofilm agent during both ARR (Wilson et al. 2017) and PTI (Xiao et al. 2024) in response to bacterial pathogens.

While the accumulation and effect of SA in leaf intercellular spaces of ARR-responding *Arabidopsis* have been well characterized, how mature healthy untreated *Arabidopsis* becomes competent to initiate the ARR response is unknown. Therefore, a transcriptomic approach was used to identify ARR-associated genes to understand how mature plants respond in a resistant manner to normally virulent *Pst*.

## Results

### Young ARR-incompetent and mature ARR-competent transcriptomes

To understand ARR competence, we performed comparative transcriptome analysis using young ARR-incompetent (3.25 wpg) and mature ARR-competent (6.25 wpg) leaves inoculated with *Pst* or mock-inoculated and collected at 15 min, 12 h, and 24 h after inoculation. At 72 h postinoculation (hpi) in mature plants, *Pst* multiplication was 250-fold lower [2.3 × 10^4^ colony-forming units (CFU)/leaf disc (ld) *Pst*] compared with young susceptible plants (5.7 ×  CFU/ld *Pst*) (Fig. S1), confirming that mature plants were highly ARR competent. After RNA was isolated and sequencing reads were processed, summarized genome-wide transcriptomic distances were compared between treatment samples to explore the expression profiles of *Pst*-inoculated leaves collected from mature and young plants. To minimize bias for genes with high expression levels, a matrix of mean transcript counts for all expressed genes was normalized by DESeq2 and log_2_-transformed. To determine the relationship between the transcriptomic profiles of each sample, pairwise Euclidean distances were calculated and the pairwise distance matrix was clustered based on average distances between clusters and visualized in a heatmap (Fig. S2a). All samples collected at 15 min postinoculation were differentiated from other samples collected at different time points, indicating that leaves collected 15 min after mock or *Pst* inoculation have a different expression profile than leaves collected at 12 or 24 hpi. At 12 and 24 hpi, treatment samples clustered predominantly by age and secondarily by inoculation time point (Fig. S2a), plus sibling nodes were differentiated by inoculation type. One exception was *Pst-*inoculated leaves from young plants collected at 24 hpi, which clustered more closely with mature mock- and *Pst*-inoculated leaves collected at 12 and 24 hpi, rather than leaves of young plants, as shown by the branches of the hierarchical tree (Fig. S2a). These data suggest that mock-inoculated leaves of mature plants are more similar to the *Pst*-responding leaves of young plants at 24 hpi than mock-inoculated leaves of young plants. This led to the idea that the leaves of mature plants may be primed to rapidly respond to *Pst.*

DESeq2 analysis was used to identify *Pst*-responsive genes (>2-fold change, adjusted *P* < 0.05) in leaves of young and mature plants. Samples were subset by time point, followed by principal component analysis of *Pst-*responsive genes to determine if the transcriptional response to *Pst* was differentiated by age and inoculum treatment at each time point. At 0.25 hpi, principal component (PC) 1 accounted for 71% of the total variance between samples and clearly distinguished young from mature samples (Fig. S2b). Similarly, PC1 accounted for 65% of the total variance at 12 hpi between samples and distinguished samples by age (Fig. S2b). At 24 hpi, 55% of the total variance (PC1) among samples distinguished mock- from *Pst*-inoculated young and mature leaves (Fig. S2b). PC2 distinguished samples by age and explained 27% of the total variability. In summary, leaf samples clustered together based on age and inoculum at each time point, and treatment groups could be differentiated from each other, demonstrating that the transcription profile of the treatments was descriptive of age and inoculum treatment for each time point.

**Figure 1:**
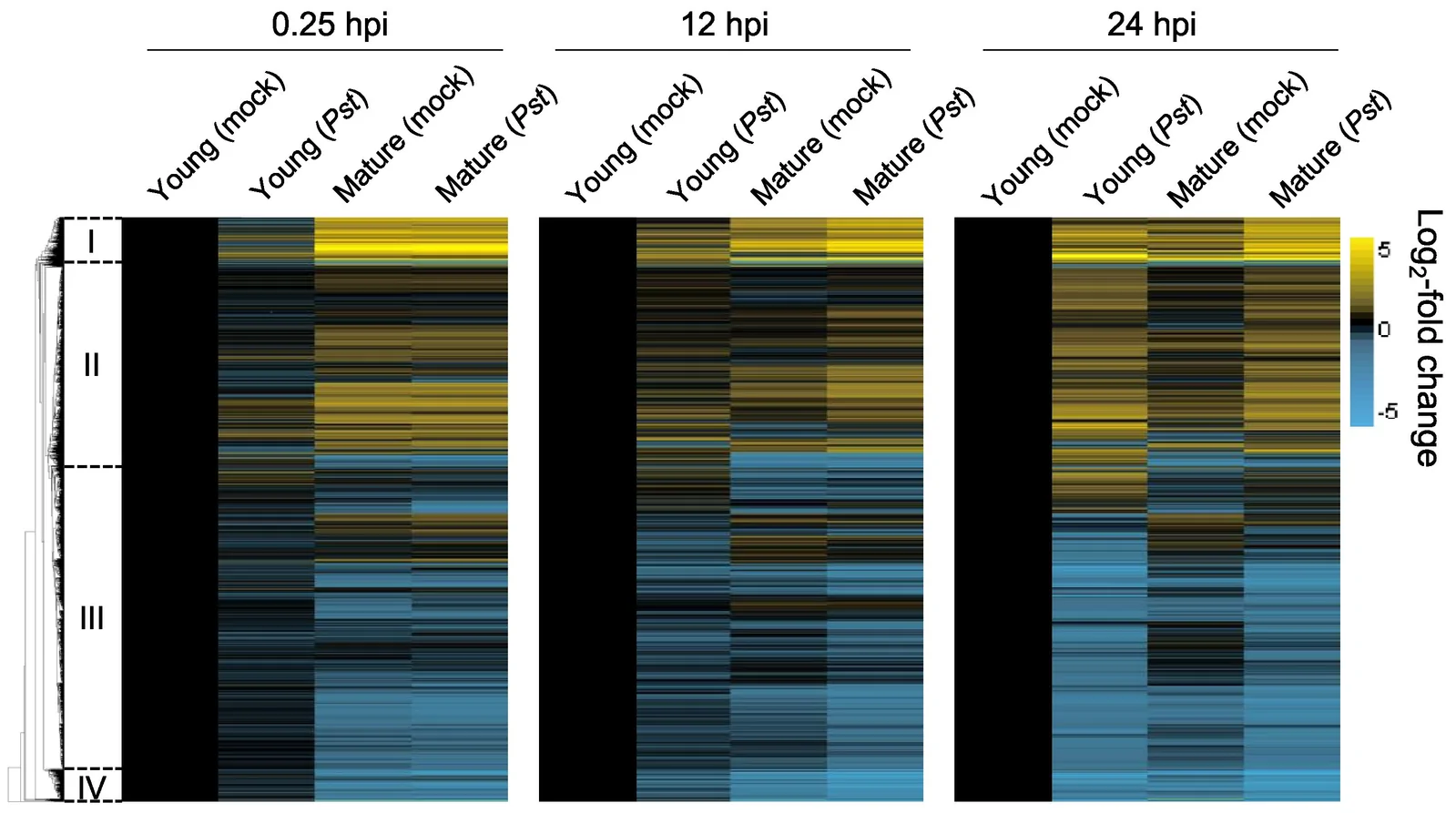
Expression of *Pst*-responsive genes in transcriptomes of young and mature Col-0 responding to *Pst*. A heatmap showing expression of *Pst*-responsive genes in Young (*Pst*), Mature (Mock), and Mature (*Pst*) relative to expression in mock-inoculated leaves of young plants at 0.25, 12, and 24 hpi (|log_2_-fold change| > 1, adjusted *P* < 0.05). A total of 6,658 genes were differentially expressed in at least one of the comparisons. Log_2_-fold changes relative to leaves of young mock-inoculated samples are shown in blue, representing a negative change, and in yellow, representing a positive change. Hierarchical clustering was used to make a tree based on the expression pattern. The tree was manually divided into 4 clades labeled I to IV based on the expression pattern.

Given that *Pst*-responsive gene expression differentiated leaf samples based on age and inoculum, mean expression of this subset of genes was used to examine how leaves of mature plants responded differently to *Pst* compared with leaves of young plants. The log_2_-fold differences relative to young mock-inoculated leaves for each gene were hierarchically clustered and produced 4 distinct clades based on the expression profile of each gene (Fig. 1). Clades II and III contained genes that were upregulated or downregulated, respectively, in leaves of both young and mature plants in response to *Pst* at 24 hpi, suggesting both clades contain common *Pst*-responsive genes. Clades I and IV contained genes that were upregulated or downregulated, respectively, in leaves of young plants at 24 hpi with *Pst* but were expressed (clade I) or repressed (clade IV) in leaves of mature plants at all time points in both mock- and *Pst*-inoculated leaves (Fig. 1). The number of genes differentially expressed in either young or mature leaves in response to *Pst* was 6,658, which is 35% of the total genes expressed in the RNA sequencing (RNA-seq) dataset (18,736 genes, >10 reads per sample) (Fig. S3). To identify genes with biologically relevant expression patterns, weighted gene correlation network analysis (WGCNA; Langfelder and Horvath 2008) clustered genes with similar expression patterns using the full dataset. The generalized expression of these clusters is summarized by sample group (eg young, mock-inoculated leaves at 24 hpi) using eigengenes (Fig. S4). Clusters 13, 36, and 37 (denoted with an asterisk in Fig. S4) contained genes expressed in leaves of young and mature plants inoculated with *Pst*, suggesting these are common *Pst*-responsive genes. Clusters 43, 45, 46, and 48 (dagger in Fig. S4) were expressed only in leaves of young susceptible plants, suggesting these clusters contain genes involved in susceptibility to *Pst* (see Materials and Methods for details).

### ARR-associated gene expression

**Figure 2:**
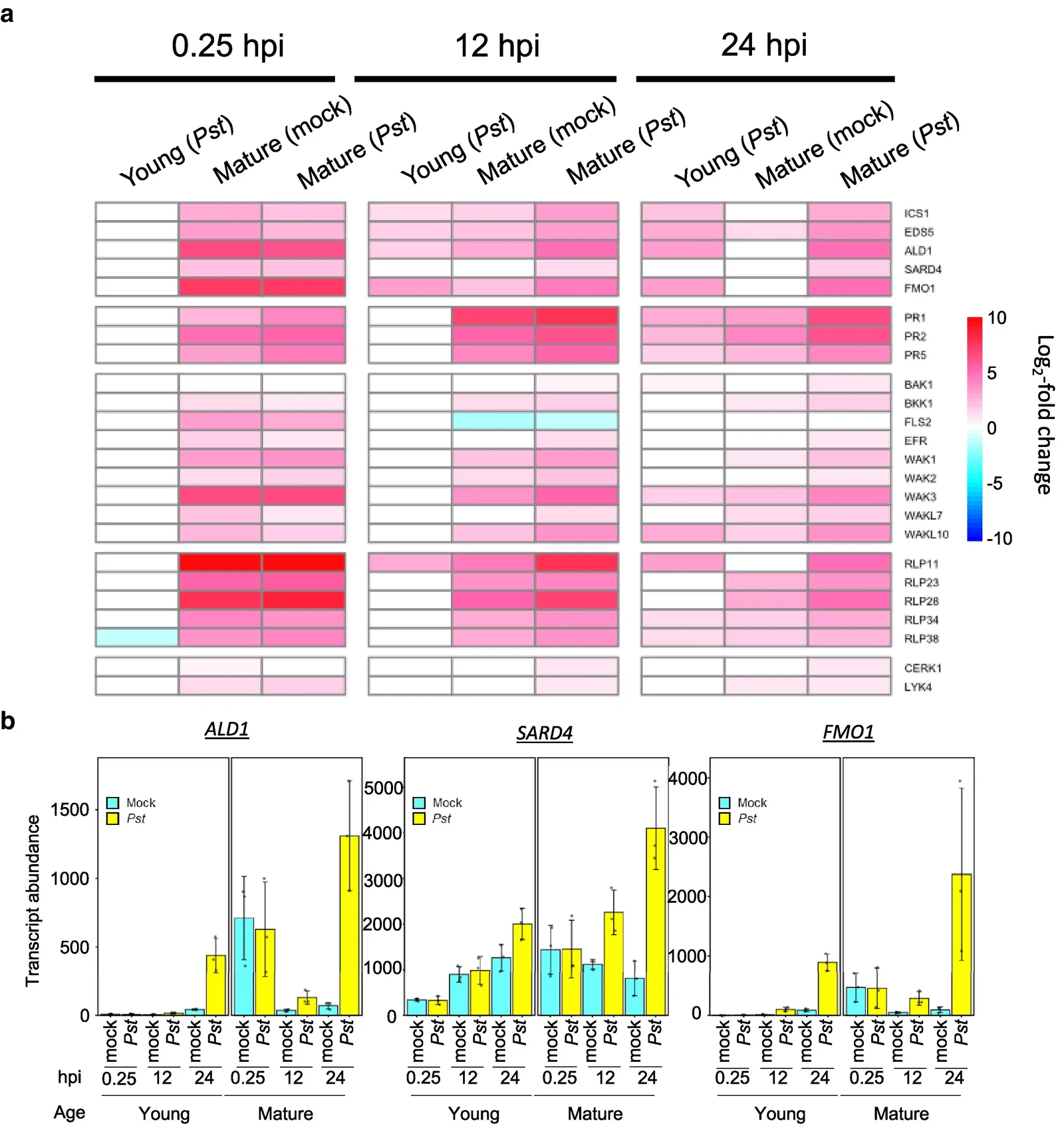
Expression of a subset of defense-related genes expressed during ARR. a) Colors represent mean log_2_-fold expression of 3 biological replicates relative to expression in the mock-inoculated leaves of young plants, where red represents significantly increased expression, blue represents significantly decreased expression, and white indicates no significant difference. b) RNA sequencing transcript abundance of NHP biosynthesis genes (*ALD1*, *SARD4*, *FMO1*) during ARR in leaves. Each bar represents the mean of 3 biological replicates, and error bars represent standard deviation.

Clusters 12, 14, and 15 (double dagger in Fig. S4) contain genes expressed more highly in leaves of mature plants responding to *Pst* compared to leaves of young *Pst*- or mock-inoculated plants, suggesting that these genes are involved in ARR. Cluster 14 contained 848 genes that were highly expressed in leaves of mature plants at 12 and 24 hpi with *Pst* compared to all other sample groups. TopGO analysis of cluster 14 revealed that these genes were associated with defense response Gene Ontology (GO) terms, including systemic acquired resistance (Fig. S5). Among these SAR GO genes were the *ALD1*, *SARD4*, and *FMO1* biosynthetic genes for the defense signal NHP (Ding et al. 2016; Hartmann et al. 2017, 2018). *PATHOGENESIS-RELATED* (*PR*) genes, *PR1*, *PR2*, and *PR5* were also upregulated in leaves of mature but not young plants in response to *Pst* at 0.25 and 12 hpi (Fig. 2), suggesting an SA-mediated defense response was activated rapidly in leaves of mature plants. *PR1*, *PR2*, and *PR5* were also expressed in mock-inoculated leaves of mature plants at 0.25 and 12 hpi but not in young mock-inoculated leaves, indicating that mature plants responded rapidly to the modest wound response that occurs during mock inoculations (Jaskiewicz et al. 2011) (Fig. 2). This suggests that mature plants were sensitized/primed to respond rapidly to wounding or *Pst*.

The WGCNA also revealed that several cell surface receptors involved in immunity were upregulated in leaves of mature plants that were mock-inoculated or inoculated with *Pst*, compared with leaves of young plants (Fig. 2). Leaves of mature plants expressed cell surface receptor genes with extracellular LYSIN motifs (LysM), EPIDERMAL GROWTH FACTOR (EGF) motifs, and LEUCINE RICH-REPEAT (LRR) motifs. *LYSM-DOMAIN CONTAINING RECEPTOR-LIKE KINASE4* (*LYK4*), involved in immunity in young plants (Wan et al. 2012), was the only LysM-type receptor upregulated in leaves of mature plants inoculated with *Pst* but not in young *Pst*-inoculated plants. Several of the EGF-type CELL WALL ASSOCIATED KINASEs and LRR receptors were also upregulated in mature mock- and *Pst*-inoculated leaves compared to young mock-inoculated leaves (Fig. 2). Many LRR-type RECEPTOR-LIKE PROTEINs (RLPs) were expressed in leaves of mature mock-inoculated compared to young mock-inoculated plants, including *RLP23* (Fig. 2), which is involved in perception of the PAMP *nlp20* (Albert et al. 2015). This analysis demonstrates that leaves of mature plants express a variety of defense-related genes in both mock- and *Pst*-inoculated leaves, supporting the hypothesis that ARR-competent leaves become sensitized or primed, allowing them to rapidly respond to *Pst*. Additional analysis of the magnitude of transcriptional responses to *Pst* in young susceptible and mature ARR-competent plants can be viewed in the Supplementary material.

### Comparison of transcriptional responses during ARR, PTI, and ETI

Given that many defense-related/SAR-associated genes were expressed in mature plants during ARR, raw sequencing data from ETI transcriptomes generated by Mine et al. (2018) were compared to PTI and ARR transcriptomes from this study to obtain insight into the similarities between these pathways. The ETI experiment by Mine et al. (2018) was chosen because Col-0 plants were grown in similar conditions as the ARR and PTI experiments, the inoculum dose was the same (106 CFU/mL *Pst avrRpm1*), and leaves were collected at the same time points (12 and 24 hpi). PTI was initiated by treating Col-0 leaves with 1 µM flg22 or sterile water (mock control). Leaves were collected for RNA-seq at 12 and 24 h posttreatment. The same pipeline as described in the Methods was used to analyze the PTI and ETI raw datasets.

**Figure 3:**
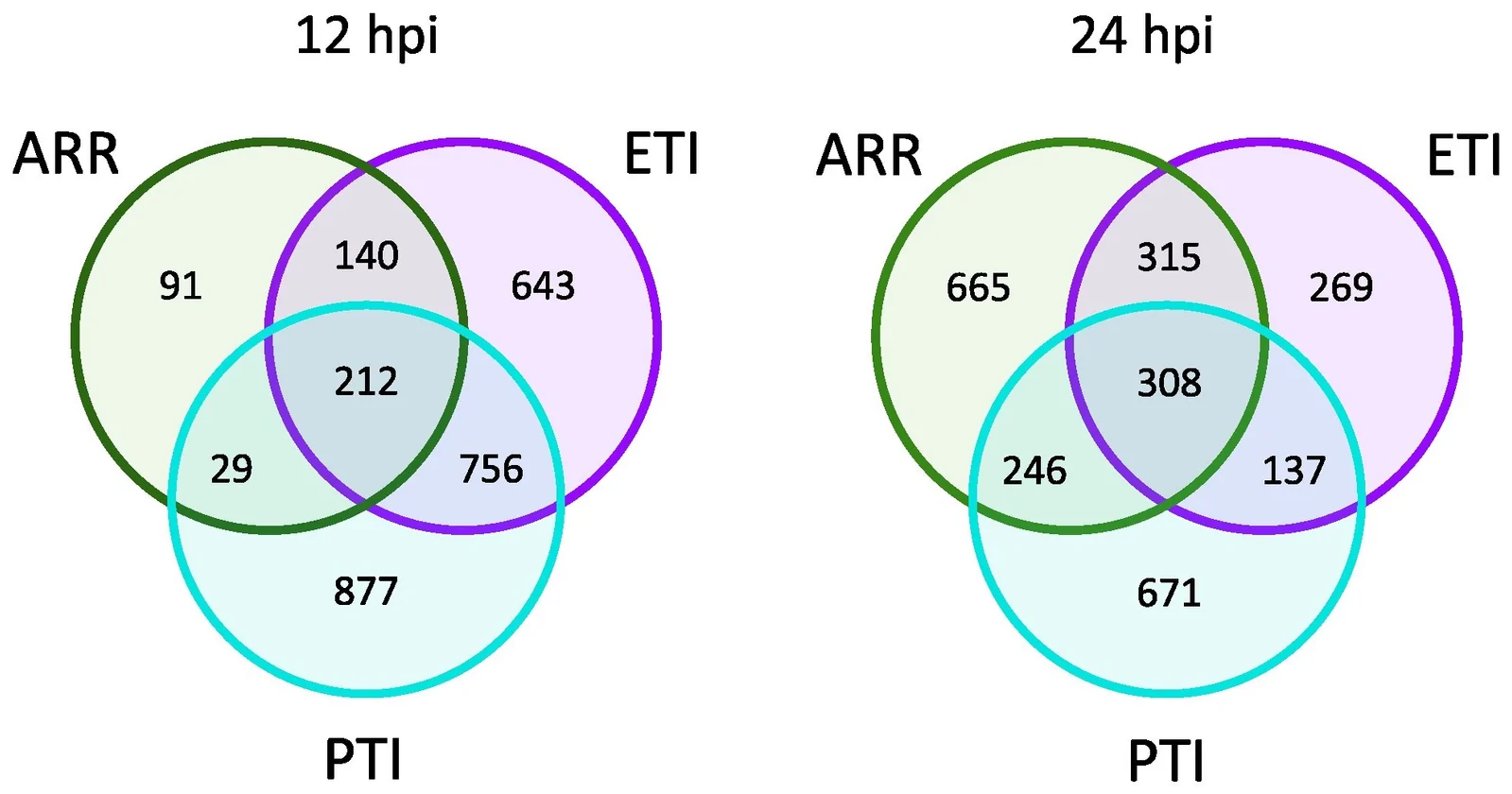
The transcriptional response of ARR compared to PTI and ETI. In each study, plants were grown under short days (9- to 10-h light days), at 22 °C, and in 60% to 80% relative humidity. PTI was elicited by treating the leaves of young plants with 1 µM flg22, a 22–amino acid epitope of flagellin from *Pst* (this work). ETI (Mine et al. 2018) was elicited by inoculating leaves of young plants with 10^6^ CFU/mL *Pst* with *avrRpt2*. ARR was initiated by inoculating mature plants with wild-type *Pst* and compared with mock-inoculated leaves of mature plants (Tables S1 and S3). Raw RNA-seq data from each study were reanalyzed using the same pipeline outlined in the methods of this study. The cutoff for differential expression was set to a fold-change >2 and adjusted *P* < 0.05. The Venn diagram displays genes significantly upregulated at 12 and 24 h postinduction of the respective immune responses.

There were 91 and 665 genes upregulated during ARR at 12 and 24 hpi, respectively, that were not upregulated in PTI or ETI (Fig. 3). These ARR-specific genes include iron uptake genes such as *FERRETIN1* (*FER1*) and *FER-LIKE IRON DEFICIENCY INDUCED TRANSCRIPTION FACTOR1* (*FIT1*) (Colangelo and Guerinot 2004; Long et al. 2008). Expression of these genes may reduce leaf intercellular iron and negatively impact *Pst* multiplication (Nobori et al. 2018). Several defense-associated genes were also upregulated in ARR-responding leaves but not in PTI or ETI, including *PR5-LIKE RECEPTOR KINASE* (*PR5K*) (Wang et al. 1996), *SUPPRESSOR OF chs1-2,3* (*SOC3*) (Zhang et al. 2017), and *PEP1 RECEPTOR2* (*PEPR2*) (Yamaguchi et al. 2010). Several auxin-related genes were upregulated during ARR but not during PTI or ETI at 24 hpi, including genes involved in the biosynthesis of auxin (see Materials and Methods for details).

Overlapping upregulated genes (adjusted *P* < 0.05, fold change >2) for PTI, ETI, and ARR were also identified (Fig. 3). Of the genes upregulated during ARR at 12 hpi, 45% (212/472) were shared with both ETI and PTI (Fig. 3). Among these co-upregulated genes were several cell surface receptors from the RLP gene family, NHP biosynthesis genes *ALD1* and *FMO1* (Ding et al. 2016; Hartmann et al. 2017, 2018), *CYTOCHROME P450 71A12* (*CYP71A12*) previously reported to contribute to ARR (Kempthorne et al. 2021), the salicylic acid biosynthesis gene *ISOCHORISMATE SYNTHASE1* (*ICS1*), and several WRKY-domain containing transcription factors (Tables S1 and S3), suggesting that PTI, ETI, and ARR use common defense genes.

SAR-associated genes were expressed in ARR, ETI, and PTI transcriptomes (Figs. 2 and 3). Moreover, PTI (via flg22; PTI dataset) and ETI (via avirulent *Pst*; Mine et al. 2018) result in the induction of SAR (Cameron et al. 1994; Mishina and Zeier 2007; Wang et al. 2019), suggesting that ARR may share signaling components with SAR. This was surprising given that *npr1-1* mutants were ARR competent (Kus et al. 2002). However, it is now known that NPR1, 3, and 4 contribute to both local PTI and ETI responses (Zhou et al. 2000; Tada et al. 2008; Ding et al. 2018), as well as SAR (Chen et al. 2018; Liu et al. 2020; Yildiz et al. 2021). This led us to investigate the involvement of SAR signaling components in ARR.

### NHP biosynthesis gene expression and accumulation during ARR

*ALD1* and *FMO1* were upregulated in ARR-responding leaves (Fig. 2), suggesting that SAR-associated NHP signaling may also contribute to ARR. NHP biosynthesis begins with the conversion of L-lysine to dehydro-pipecolic acid by the aminotransferase ALD1 (Hartmann et al. 2017). Although not absolutely required, SAR-DEFICIENT4 (SARD4) catalyzes the reduction of dehdyro-pipecolic acid to pipecolic acid (Ding et al. 2016), which is hydroxylated to NHP by the FMO1 protein (Hartmann et al. 2018). To validate the RNA-seq data, *ALD1* and *FMO1* expression was examined in leaves collected from young susceptible and mature ARR-competent plants that were mock-inoculated or inoculated with 10^6^ CFU/mL *Pst* at 6 and 12 hpi. Bacterial levels were quantified at 72 hpi, confirming that mature plants displayed a strong ARR response (350-fold less *Pst* in mature compared to young leaves; Fig. S6). Since *ALD1* and *FMO1* were expressed in mature mock-inoculated plant transcriptomes at 0.25 hpi (Fig. 1), we speculated that leaves of mature plants may be expressing *ALD1* and *FMO1* prior to inoculation. Therefore, untreated leaves from young and mature plants were also collected.

**Figure 4:**
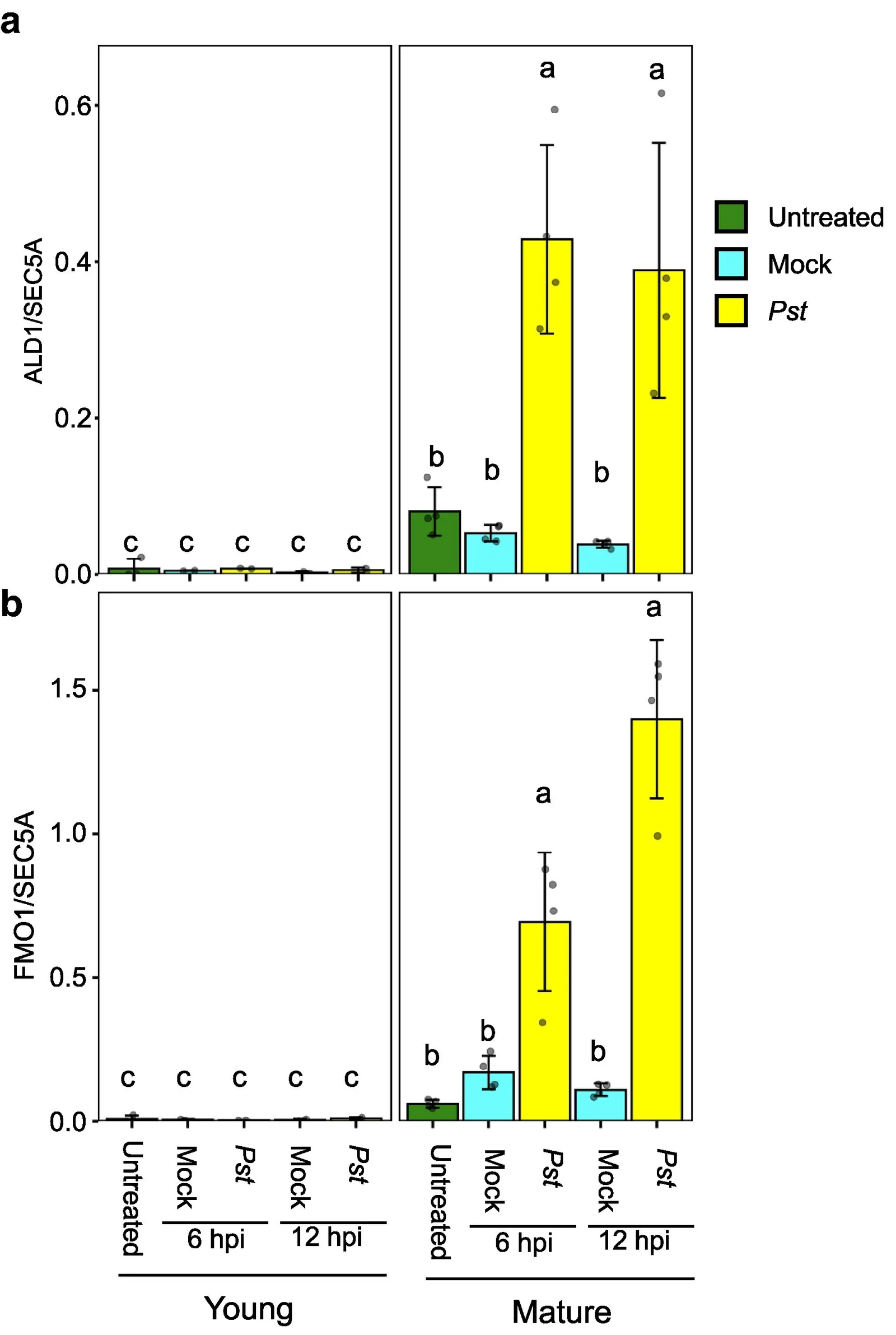
*ALD1* and *FMO1* expression in young and mature plants. At 3.25 and 6.25 wpg, leaves were inoculated with 10^6^ CFU/mL *Pst* or mock-inoculated (10 mM MgCl_2_). At 72 hpi, bacterial levels were quantified (Fig. S6). RNA was extracted from untreated, mock-inoculated, or *Pst*-inoculated leaves at the specified time points. Quantitative PCR was used to examine gene expression of *ALD1* (a) and *FMO1* (b) relative to the reference genes *SEC5A* (Fig. 4) or *CUL4* (Fig. S12). Outliers were calculated using the 1.5× interquartile range (IQR) method and depicted as triangles. Each bar represents the mean of 2 to 4 biological replicates, and error bars represent the standard deviation. Different letters indicate statistically significant differences (ANOVA, Tukey's honestly significant difference, *P* < 0.05). This experiment was completed 2 additional times with similar results.

*ALD1* (58-fold) and *FMO1* (130-fold) were highly expressed in mature leaves of untreated ARR competent compared to untreated leaves of young plants (Fig. 4). Distant immune-ready leaves of SAR-induced plants express *ALD1* and *FMO1* (Návarová et al. 2012; Bernsdorff et al. 2016) to similar levels as mature ARR-competent plants, suggesting that ARR may include a defense priming or immune-ready stage. Similar to the literature (Mine et al. 2018) and the RNA-seq data (Fig. 2b), little expression of *ALD1* and *FMO1* was observed in young mock- and *Pst*-inoculated leaves at 6 and 12 hpi (Fig. 4). However, in mature plants, *ALD1* was upregulated in *Pst-*inoculated compared to mock-inoculated leaves at both 6 and 12 hpi by 8- and 10-fold, respectively (Fig. 4a). *FMO1* was also upregulated in *Pst*-inoculated compared to mock-inoculated leaves of mature plants at 6 and 12 hpi by 4- and 13-fold, respectively (Fig. 4b), indicating NHP biosynthesis gene expression is rapidly upregulated in leaves of mature plants responding to *Pst*. Rapid and strong expression of *ALD1* and *FMO1* following inoculation with *Pst* during ARR in mature plants (Fig. 4) is similar to what is observed during SAR in distant immune-ready leaves in response to a subsequent pathogen attack (Návarová et al. 2012; Bernsdorff et al. 2016), supporting the idea that untreated leaves of mature plants are primed/immune ready.

### Functional NHP biosynthesis genes are required for ARR

**Figure 5:**
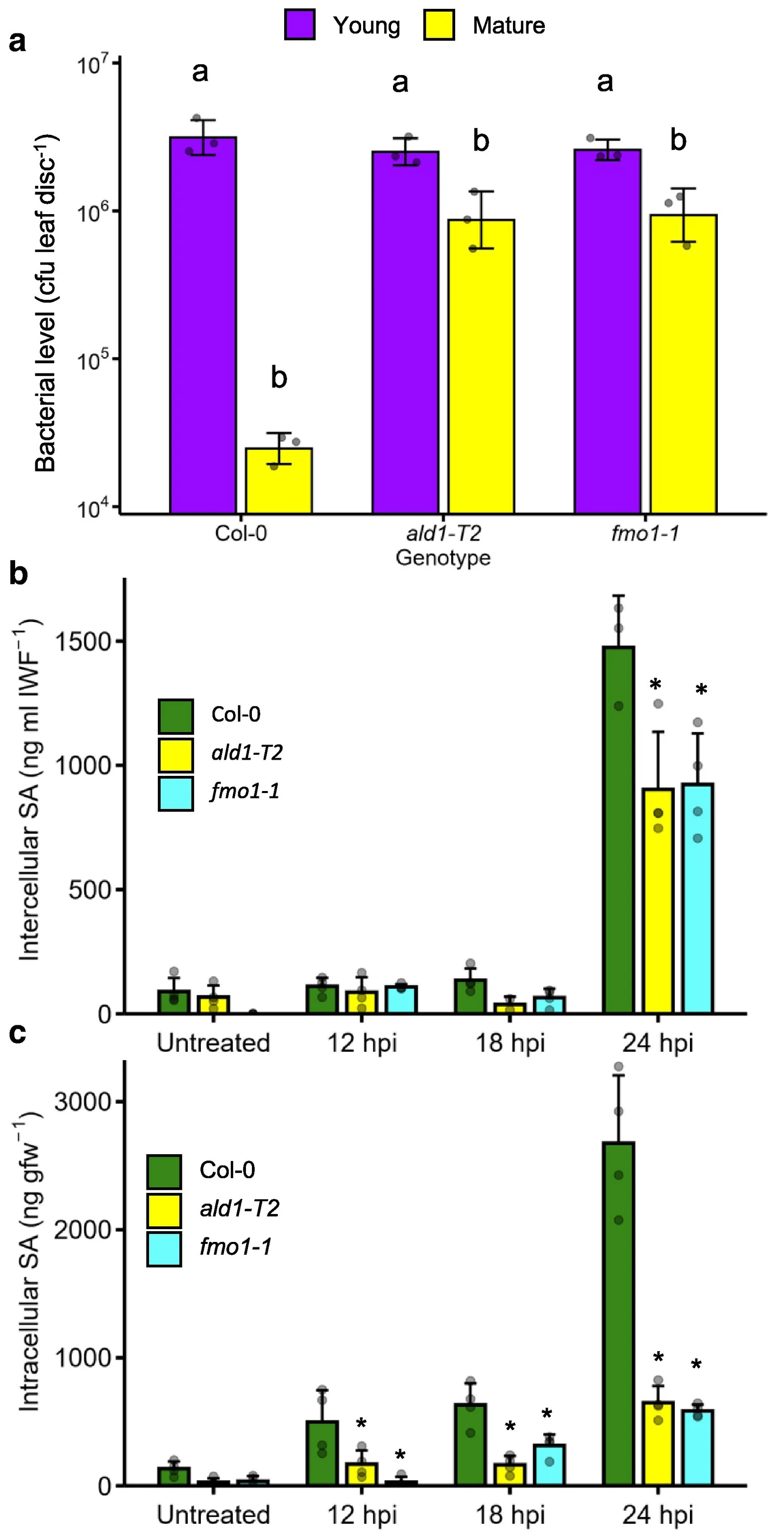
NHP biosynthesis gene function contributes to ARR and SA accumulation in mature Col-0, *ald1-T2*, and *fmo1-1* responding to *Pst.* a) Leaves were inoculated with 10^6^ CFU/mL *Pst* at 3.25 or 6.25 wpg, and bacterial levels were quantified at 72 hpi. Each bar represents the mean of 3 biological replicates displayed on a logarithmic y-axis, and error bars show the standard deviation. This experiment was completed 5 times with similar results (Fig. S7). b and c) Intercellular washing fluids (IWFs) were collected from leaves of mature plants that were untreated or inoculated with 10^6^ CFU/mL *Pst*. SA was quantified in IWFs (b) or corresponding leaf tissue minus IWFs (c) using an SA biosensor. Asterisks indicate significant differences between genotypes at the same time point (*t* test, *P* < 0.05). Each bar shows the mean of 4 biological replicates, and error bars show the standard deviation. This experiment was completed one additional time with similar results observed.

To investigate if NHP biosynthesis is required for the ARR response, ARR was examined in Col-0, *ald1-T2* mutants unable to produce dehydro-pipecolic acid, pipecolic acid, or NHP (Ding et al. 2016; Hartmann et al. 2017) and *fmo1-1* mutants, which produce pipecolic acid, but not NHP (Hartmann et al. 2018). Young Col-0, *ald1-T2*, and *fmo1-1* leaves were similarly susceptible to infection (Fig. 5a). A strong ARR response was observed in mature Col-0, as demonstrated by 130-fold lower *Pst* levels in mature compared with young Col-0 leaves, whereas mature *ald1-T2* and *fmo1-1* were compromised for the ARR response as they supported high levels of *Pst* compared with Col-0. However, *ald1-T2* and *fmo1-1* were not entirely ARR defective as mature leaves supported 3-fold lower *Pst* compared with young leaves (Fig. 5a). These data provide evidence that functional NHP biosynthesis genes are required for ARR and suggest that NHP biosynthesis is important for ARR. In response to *P. syringae* pv. *maculicola*, the *sard4-5* mutant accumulates less pipecolic acid [∼13 μg/gram fresh weight (gfw)] compared with Col-0 (∼25 to 30 μg/gfw) (Ding et al. 2016). Therefore, ARR was also examined in *sard4-7* (SALK_053625C) to determine if reduced pipecolic acid production affects the ARR response. Mature *sard4-7* leaves displayed an intermediate ARR response between fully ARR-competent Col-0 and ARR-defective *ald1-T2* and *fmo1-1* (Fig. S7), suggesting that SARD4 contributes to but is not essential for ARR.

If NHP contributes to ARR signaling for intercellular SA accumulation, a key downstream component of ARR (Cameron and Zaton 2004; Wilson et al. 2017), SA accumulation should be reduced in the ARR-defective NHP biosynthesis mutants, *ald1-T2* and *fmo1-1* (Fig. 5a). Intercellular SA accumulation was examined in the ARR experiment shown in Fig. 5a by collecting intercellular washing fluids (IWFs) from mature leaves before and after inoculation with *Pst*. Similar low levels of intercellular SA (<100 ng/mL IWF) were observed in IWFs collected from leaves of mature untreated Col-0, *ald1-T2*, and *fmo1-1*, as well as in IWFs from Col-0, *ald1-T2*, and *fmo1-1* at 12 and 18 hpi with *Pst* (Fig. 5b). At 24 hpi, Col-0 IWFs contained ∼1,470 ng/mL SA (Fig. 5b), while *ald1-T2* and *fmo1-1* IWFs contained significantly less SA (∼900 and 920 ng/mL IWF, respectively) (Fig. 5b). Therefore, in response to *Pst*, mature NHP biosynthesis mutants accumulated less intercellular SA compared with wild-type Col-0 (Fig. 5b), and this may contribute to the reduced ARR response observed in *ald1-T2* and *fmo1-1* (Fig. 5a).

Before SA accumulates in the intercellular space, it is produced inside plant cells; thus, intracellular SA levels were also determined in corresponding leaves after IWF removal to determine if intracellular SA biosynthesis was reduced in *ald1-T2* and *fmo1-1* leaf cells. Wild-type Col-0 intracellular SA levels in untreated leaves were low (∼100 ng SA/gfw) as expected (Carviel et al. 2014), with greater SA accumulation at 12 and 18 hpi (∼500 ng/gfw) and 24 hpi (2,500 ng/g FW) (Fig. 5c). At 18 hpi, *ald1-T2* and *fmo1-1* leaves accumulated ∼2-fold less intracellular SA (160 and 300 ng/g FW, respectively) than Col-0 and ∼4.5-fold less at 24 hpi (650 and 580 ng/gfw, respectively) (Fig. 5c). In response to *Pst*, both *ald1-T2* and *fmo1-1* accumulated less inter- and intracellular SA compared to wild-type (Fig. 5a and b), suggesting that NHP signaling during ARR is important for downstream SA accumulation.

### *ALD1* and *FMO1* expression are associated with ARR competence

**Figure 6:**
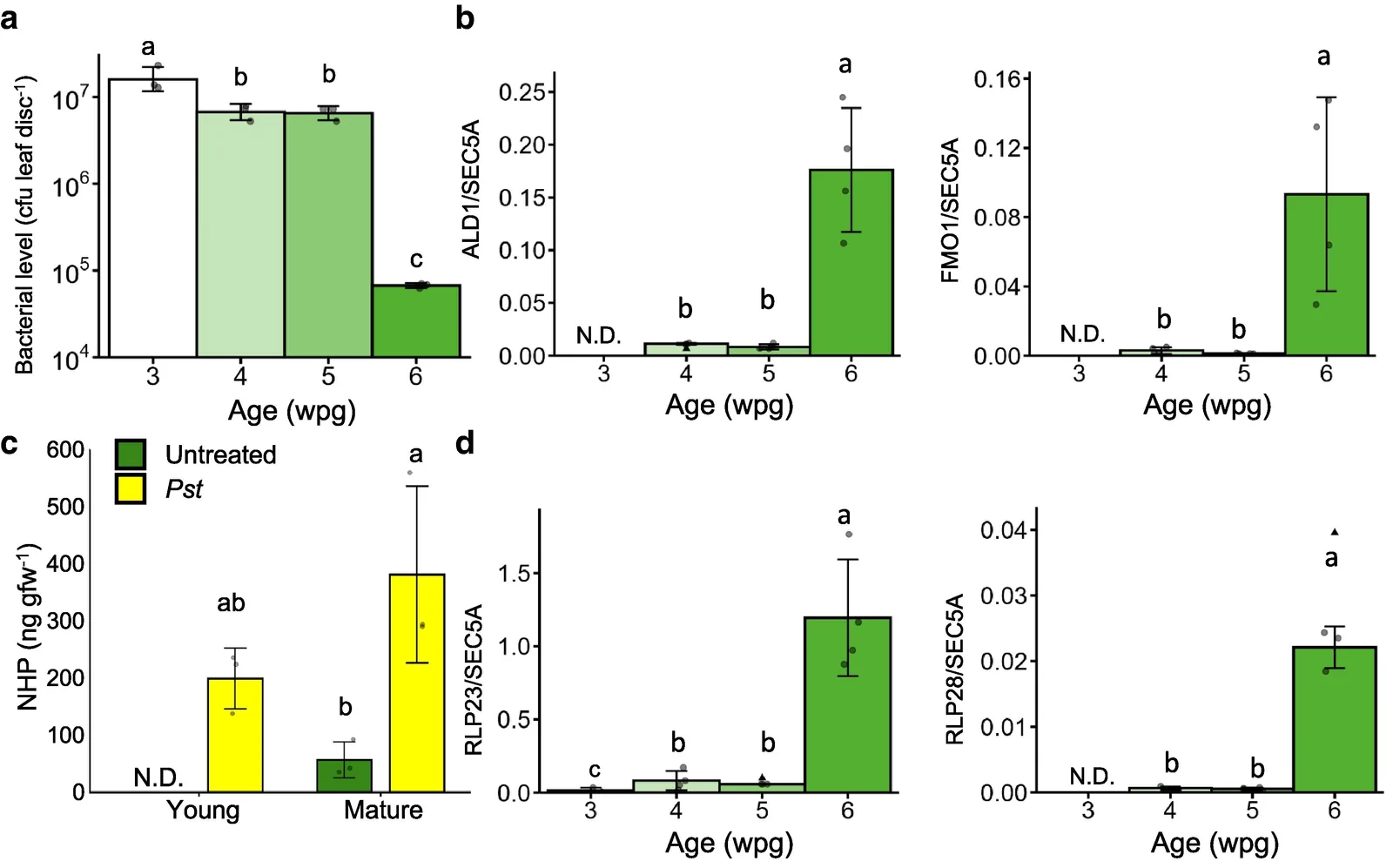
NHP biosynthesis gene and cell surface receptor gene expression associated with ARR onset. Leaves were inoculated each week with 10^6^ CFU/mL *Pst* until the onset of ARR at 6 wpg. a) Bacterial levels were quantified 72 hpi. Each bar represents the mean of 3 biological replicates displayed on a logarithmic y-axis, and error bars show standard deviation. b and d) Prior to inoculation, leaf tissue was collected for RNA extraction. Quantitative PCR was used to examine gene expression relative to the reference genes *SEC5A* (this figure) or *CUL4* (Fig. S13). Outliers were calculated using the 1.5× IQR method and depicted as triangles. Each bar represents the mean of at least 3 biological replicates, and error bars represent the standard deviation. Different letters indicate statistically significant differences (ANOVA, Tukey's honestly significant difference, *P* < 0.05). Expression of ALD1, FMO1, and RLP28 was not detected (N.D.) in 3 wpg samples. This experiment was completed 2 additional times with similar results. c) The leaves of young 3.5 wpg and mature 6.5 wpg plants were either left untreated or inoculated with 10^6^ CFU/mL *Pst*. At 24 hpi, leaf tissue was collected for NHP extraction. LC-MS/MS was used to quantify the amount of NHP in each sample. Samples in which NHP was not above the detection threshold are denoted as not detected (N.D.). Each bar represents the mean of at least 3 biological replicates, and error bars represent the standard deviation. Different letters indicate statistically significant differences (ANOVA, Tukey's honestly significant difference, *P* < 0.05). This experiment was completed 2 additional times with similar results.

NHP signaling may contribute to establishing ARR competence or, in other words, a primed/immune ready state in healthy mature plants, as *ALD1* and *FMO1* were expressed in untreated leaves of mature plants (Fig. 4). To investigate if *ALD1* and *FMO1* expression is associated with ARR-competence, which occurs at ∼6 wpg under short-day conditions, *ALD1* and *FMO1* expression levels were examined in healthy untreated Col-0 leaves at 3, 4, 5, and 6 wpg. The ARR response was tracked by measuring *Pst* multiplication in leaves at 3, 4, 5, and 6 wpg. Plants were highly susceptible, as demonstrated by high *Pst* levels at 3 wpg (2 × 10^7^ CFU/ld) and 4 and 5 wpg (∼6.5 × 10^6^ CFU/ld) (Fig. 6a). Plants responded with a strong ARR response, as demonstrated by a 100-fold reduction in *Pst* (10^5^ CFU/ld) at 6 wpg compared to 3 wpg (Fig. 6a). *ALD1* and *FMO1* expression in untreated leaves remained low at 3, 4, and 5 wpg compared to 6 wpg (Fig. 6b). At 6 wpg, *ALD1* and *FMO1* were expressed at 10-fold higher levels compared with 5 wpg (Fig. 6b), suggesting that NHP biosynthesis gene expression at 6 wpg may be involved in creating an ARR immune ready/primed state.

Metabolites were extracted from untreated and *Pst*-inoculated leaves at 24 hpi from young and mature plants to determine if NHP accumulation is associated with ARR competence. NHP was quantified by liquid chromatography/tandem mass spectrometry (LC-MS/MS) using a polar reverse-phase column and a triple quadrupole mass spectrometer with 2-hydroxycyclohexane carboxylic acid as an internal standard. Like other studies (Hartmann et al. 2018; Cai et al. 2021), NHP was not detected in untreated leaves of young plants but was detected at 24 hpi with *Pst* (200 ng/gfw) (Fig. 6c). Untreated leaves of ARR-competent plants accumulated 55 ng/gfw of NHP and 380 ng/gfw of NHP in response to *Pst* at 24 hpi (Fig. 6c). Accumulation of NHP in untreated leaves of mature ARR-competent plants provides support for the idea that similar to priming during SAR (Cai et al. 2021), mature Arabidopsis plants become primed/immune ready, allowing them to respond with ARR to normally virulent *Pst*.

### Cell surface receptors are required for ARR

The ARR RNA-seq data analysis also identified many genes in the plasma membrane localization GO term (Fig. S5), including 20 leucine-rich RLPs (Fig. S8; Hooper et al. 2017), which were expressed 15 min after mock or *Pst* inoculation in mature plants (Fig. S8). The RLP23 receptor is involved in perception of the damage-associated peptide nlp20 (Böhm et al. 2014; Albert et al. 2015), and *RLP28* is implicated in salt stress tolerance (Wu et al. 2016). Given that *RLP23* and *RLP28* were highly upregulated in ARR-competent mature leaves compared with young plants responding to *Pst* (Fig. 2a), expression of RLP23 and RLP28 was examined in healthy untreated leaves at 3, 4, 5, and 6 wpg. Little *RLP23* and *RLP28* expression was observed at 3, 4, and 5 wpg in healthy untreated leaves, but at 6 wpg, both *RLP23* and *RLP28* were expressed (Fig. 6d), suggesting these cell surface receptors are also associated with the ARR competent primed/immune ready state in mature plants.

**Figure 7:**
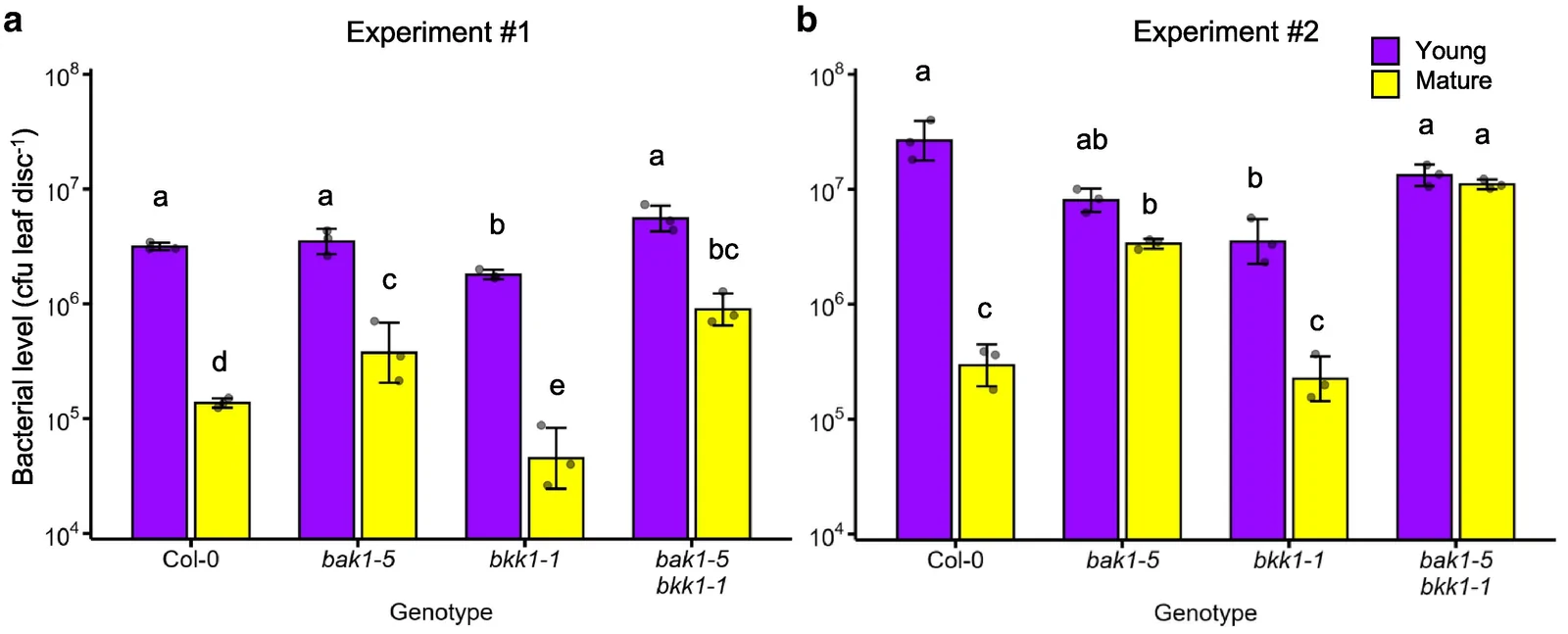
The cell surface coreceptors BAK1 and BKK1 are important for ARR. Col-0, *bak1-5, bkk1-1*, and *bak1-5 bkk1-1* leaves were inoculated with 10^6^ CFU/mL *Pst* at 3.5 (young) or 6.5 (mature) wpg, and bacterial levels were quantified at 72 hpi. Each bar represents the mean of 3 biological replicates displayed on a logarithmic y-axis, and error bars show the standard deviation for experiments 1 and 2 (ANOVA, Tukey's honestly significant difference, *P* < 0.05).

Rather than investigating all 20 RLPs upregulated during ARR (Fig. S8), the downstream coreceptors BRI1-ASSOCIATED RECEPTOR KINASE1 (BAK1) and BAK1-LIKE1 (BKK1) were investigated. BAK1 and BKK1 redundantly associate with many receptors involved in perceiving pathogen- and damage-associated molecular patterns and are required to transduce cell surface receptor/ligand interactions, including FLS2/flg22 and RLP23/nlp20 (Chinchilla et al. 2007; Albert et al. 2015). The ARR phenotypes of *bak1-5*, *bkk1-1*, and the *bak1-5 bkk1-1* double mutants were examined to determine if pathogen perception by BAK1/BKK1-requiring receptors is involved in the ARR response to *Pst*. The *bkk1-5* mutant was ARR competent in experiments 1 and 2, while the *bak1-1* mutant was partially defective for ARR in both experiments (Fig. 7). The *bak1-5 bkk1-1* double mutant displayed a partial ARR defect in experiment 1 (Fig. 7a) and a full ARR defect in experiment 2 (Fig. 7b), suggesting that cell surface signaling mediated by BAK1 and, to a lesser extent, by BKK1 is required for a full ARR response.

### ARR and SAR transcriptome comparative analysis

Given that ARR appears to be similar to SAR, a SAR transcriptome (Baum et al. 2019) was compared to ARR transcriptomes from this study to obtain additional insight into the similarities between SAR and ARR. In young plants, SAR is induced by infection in 1 leaf, resulting in movement of mobile signals via the phloem to distant leaves, where the signals are perceived and the primed/immune ready state is established. This allows the distant leaves to respond to normally virulent pathogens with resistance, referred to as the SAR manifestation stage (Champigny and Cameron 2009). Before this study, our accumulated data suggested that ARR is not similar to SAR; therefore, transcriptomes from untreated leaves of mature plants that represent the primed/immune ready stage of ARR were not obtained. However, ARR transcriptomes obtained in this study represent the manifestation stage of ARR (mock- or *Pst* challenge-inoculated leaves of mature plants), as supported by Figs. 1 to 7. Therefore, we compared our ARR manifestation transcriptomes to the Baum et al. (2019) SAR manifestation dataset, in which local leaves of young plants were mock-induced or SAR-induced (3 × 10^8^ CFU/mL *Psm*), followed by challenge of distant leaves 72 h later with a second stress (infiltration with tap water) and leaf collection at 2.5 hpi (early SAR manifestation stage). Given that the 0.25 hpi ARR transcriptome represents very early expression in the ARR manifestation stage and the 24 hpi ARR transcriptome time point does not include the early to mid-ARR manifestation stage, the 12 hpi ARR manifestation time point was selected for the comparative analysis. Mock-inoculated leaves of young plants (this study) were used as the unprimed control for comparison, with the ARR manifestation stage represented by mature mock-inoculated (Fig. S9a) and mature *Pst*-inoculated (Fig. S9b).

In comparing the ARR and SAR manifestation datasets, 36% of genes (467/1,291) were upregulated in all 3 datasets, suggesting a common set of genes for both ARR and SAR manifestation stages (Fig. S9). ARR-associated genes identified in Fig. 2 were also observed in the ARR/SAR manifestation genes (Fig. S9), including *RLP11/23/28/34*, *EDS5*, *ICS1*, *FMO1*, and *PR1/2/5*. Genes in all 3 manifestation datasets included the SAR master regulators *CBP60g* and *SARD1* (Shields et al. 2022) and *CYP71A13* (Kempthorne et al. 2021), which were not identified in Fig. 2. A number of genes were upregulated during ARR manifestation to *Pst* and during SAR manifestation (Baum et al. 2019) but not during ARR manifestation to mock inoculation (Table S2), suggesting that ARR- and SAR-primed plants respond differently to challenge with a pathogen compared with a mock challenge. These pathogen-specific manifestation genes included the cell surface receptors *ADR1*, *ADR1-L1/2*, and *NPR3/4.* Many genes common to both the ARR and SAR manifestation stages were identified in this comparative analysis, supporting the idea that the ARR response is similar to SAR.

### Functional *NPR1* and *NPR4* are required for ARR

NPR1 was thought to be unnecessary for ARR as mature *npr1-1* plants were shown to display an ARR response (Kus et al. 2002). However, the involvement of NPR proteins in NHP-mediated SAR (Liu et al. 2020) and our data on NHP involvement in ARR (Figs. 4 to 6) led us to reexamine the role of NPR1 and NPR4 during ARR. NPR1 (Wu et al. 2012) and NPR4 (Fu et al. 2012) are both SA receptors but have opposing roles in transcriptional regulation during plant immune responses (Ding et al. 2018). At low concentrations of SA, NPR1 is in an inactive oligomer in the cytosol (Mou et al. 2003), and NPR4 acts in the nucleus as a transcriptional repressor of SA-responsive genes (Ding et al. 2018) and as an E3 ligase to mediate degradation of NPR1 (Fu et al. 2012). As the concentration of SA increases, NPR4 and NPR1 bind to SA, which leads to inactivation of NPR4 and activation of NPR1 (Fu et al. 2012; Liu et al. 2020; Wang et al. 2020), which is thought to be caused by redox changes due to higher cytosolic SA concentrations, leading to monomerization of NPR1 (Tada et al. 2008) and translocation of NPR1 into the nucleus (Kinkema et al. 2000), where it binds and activates TGA transcription factors (Rochon et al. 2007), which control a variety of defense genes during PTI (Liu et al. 2020), ETI (Chen et al. 2017), and SAR (Ding et al. 2020).

**Figure 8:**
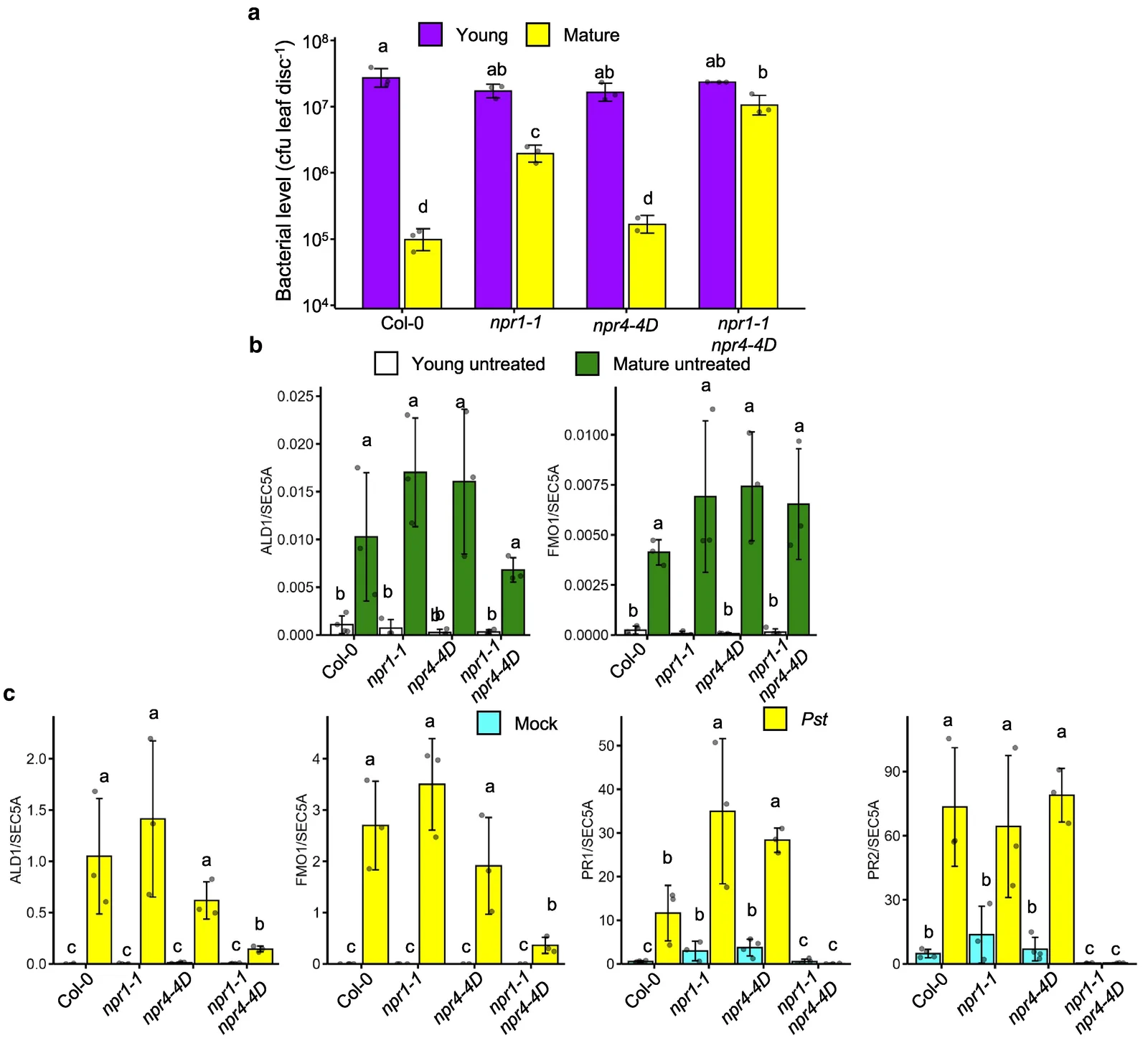
NPR1 and NPR4 effects on gene expression during ARR. a) Leaves of Col-0, *npr1-1, npr4-4D*, and *npr1-1 npr4-4d* were inoculated with 10^6^ CFU/mL *Pst* at 3.25 (young) or 6.25 (mature) wpg and bacterial levels quantified at 72 hpi. Each bar represents the mean of 3 biological replicates displayed on a logarithmic y-axis, and error bars show standard deviation. Different letters indicate statistically significant differences (ANOVA, Tukey's honestly significant difference, *P* < 0.05). This experiment was completed 3 times in total with similar results observed (Fig. S14). b and c) RT-qPCR expression data were obtained from untreated young and mature leaves (b) and at 24 hpi from leaves of mature plants inoculated with 10^6^ CFU/mL *Pst* or mock-inoculated with 10 mM MgCl_2_ (c). Gene expression was quantified relative to the reference gene *SEC5A* (this figure) and *CUL4* (see raw data file link in the Materials and Methods). Bars depict the mean of 3 to 4 biological replicates, and error bars show the standard deviation. Different letters indicate statistically significant differences (ANOVA, Tukey's honestly significant difference, *P* < 0.05).

The loss-of-function *npr1-1* mutant is not completely defective in SA signaling (Cao et al. 1997; Ding et al. 2020), and the gain-of-function *npr4-4D* mutant encodes an *npr4* allele that lacks the ability to bind SA and therefore constitutively represses SA-mediated gene expression and signaling (Ding et al. 2018), such that *npr1-1 npr4-4D* double mutants are completely SA-insensitive with no downstream SA signaling and are highly defective for PTI, ETI, and SAR (Liu et al. 2020). Therefore, the *npr1-1 npr4-4D* double mutant was used to investigate if SA-mediated NPR1 signaling is required for ARR. An ARR assay was performed with *npr1-1*, *npr4-4D*, and the *npr1-1 npr4-4D* double mutant. As observed in past studies (Liu et al. 2020), the leaves of young Col-0 *npr1-1*, *npr4-4D*, and *npr1-1 npr4-4D* plants were similarly susceptible to *Pst*. A strong ARR response was observed in mature compared with young Col-0 (280-fold lower *Pst* levels) (Fig. 8a; Fig. S14), whereas the leaves of mature *npr1-1* plants were ∼10-fold more susceptible to *Pst* than leaves of mature Col-0 (Fig. 8a), suggesting that functional NPR1 is required for a full ARR response. Meanwhile, the leaves of mature *npr4-4D* plants displayed a full ARR response (Fig. 8a), suggesting the *npr4-4D* mutation has no effect on ARR in plants with a functional copy of NPR1. High bacterial levels (>10^7^ CFU/ld) were observed in leaves of young and mature *npr1-1 npr4-4D* (Fig. 8a), indicating that *npr1-1 npr4-4D* was fully ARR defective, suggesting that SA-mediated signaling by NPR1 and NPR4 contributes to ARR.

Given that SA-mediated NPR1 signaling is involved in ARR (Fig. 8) and contributes to NHP biosynthesis during SAR in young plants (Liu et al. 2020), the role of NPR1- and NHP-mediated signaling during ARR was explored by examining the expression of *ALD1* and *FMO1* using quantitative reverse transcription PCR (RT-qPCR) in *npr1-1 npr4-4D.* Untreated leaves of mature Col-0, *npr1-1, npr4-4D*, and *npr1-1 npr4-4D* consistently expressed *ALD1* and *FMO1* to similar levels (>10-fold) compared with untreated young leaves (Fig. 8b). This suggests that SA-mediated NPR1 signaling is not required to regulate NHP biosynthesis genes *ALD1* and *FMO1* in mature immune-ready/primed untreated plants and that NHP acts upstream of SA-mediated NPR1 signaling.

While *ALD1* and *FMO1* expression was similar in untreated leaves of mature *npr* mutants and Col-0, NPR1-mediated signaling may regulate NHP biosynthesis genes during ARR in response to *Pst.* Therefore, RT-qPCR was performed with mock- and *Pst*-inoculated leaves of mature plants at 24 hpi. *ALD1* and *FMO1* were expressed (500- to 5,000-fold) in *Pst-*inoculated leaves compared with mock-inoculated leaves of Col-0, *npr1-1*, and *npr4-4D* (Fig. 8c), indicating that *npr1-1* and *npr4-4D* mutants display wild-type expression of NHP biosynthesis genes during ARR. *Pst*-inoculated leaves of the *npr1-1 npr4-4D* double mutant expressed *ALD1* and *FMO1* less than wild-type *Pst*-inoculated leaves (5- and 4-fold reduction, respectively; Fig. 8c), suggesting that SA-mediated NPR1 signaling is required for the expression of NHP biosynthesis genes during the ARR response. *Pst*-inoculated leaves of mature *npr1-1 npr4-4D* plants expressed *ALD1* and *FMO1* to higher levels compared with mock-inoculated leaves (63- and 855-fold, respectively; Fig. 8c), suggesting that an NPR1-independent mechanism also contributes to *ALD1* and *FMO1* expression during ARR.

Given that NPR1/4-mediated signaling is important for ARR (Fig. 8a) and for SAR, PTI, and ETI (Zhou et al. 2000; Tada et al. 2008; Ding et al. 2018; Liu et al. 2020), downstream PR gene expression was examined during ARR in mature plants. *PR1* and *PR2* were expressed from 7- to 20-fold higher in *Pst*-inoculated leaves of Col-0, *npr1-1*, and *npr4-4D* compared with mock-inoculated leaves (Fig. 8c), suggesting that loss of NPR1 or NPR4 does not negatively impact *PR1* and *PR2* gene expression in mature leaves during ARR. However, *PR1* and *PR2* genes were not expressed in mature leaves of *npr1-1 npr4-4D* in response to *Pst* (Fig. 8c), suggesting that SA-mediated NPR1/4 signaling is important for the expression of *PR* genes during the ARR response and that PR1 and PR2 are important for ARR.

## Discussion

### ARR includes an immune-ready/primed/ARR-competent state similar to SAR

Comparative transcriptome analysis of young susceptible and mature ARR-responding plants identified many ARR-associated genes that are also associated with PTI, ETI, and the primed/immune ready state in systemic leaves during SAR. For example, *Psm*-induced SAR resulted in the expression of 70% (37/53) of RLPs in distant uninoculated systemic leaves (Bernsdorff et al. 2016), and *RLP23* and *RLP28* expression was upregulated in leaves treated with 1 mM NHP (Yildiz et al. 2021). Similarly, ARR was associated with the expression of 44% (20/45) of RLPs. Moreover, *RLP23* and *RLP28* were expressed in ARR-competent healthy leaves and in response to *Pst* during ARR. Similar to distant primed leaves during SAR (Cai et al. 2021), expression of the NHP biosynthesis genes *ALD1* and *FMO1* was observed in ARR-competent healthy leaves, providing support for the existence of an ARR immune-ready/primed state that is similar to SAR. We hypothesize that plants become developmentally primed/immune ready for ARR between 5 and 6 wpg because untreated leaves of 5-week-old plants expressed very low levels of NHP biosynthesis genes and were ARR defective, while untreated leaves of 6-week-old plants expressed NHP biosynthesis genes, accumulated NHP, and displayed ARR in response to *Pst*.

Mature plants may become primed/immune ready for ARR between 5 and 6 weeks of age, as a result of epigenetic changes that lead to chromatin opening at key SAR/ARR gene promoters, as has been observed in systemic leaves during SAR (Baum et al. 2019). Or perhaps ARR priming uses a similar epigenetic mechanism as jasmonic acid (JA)-dependent long-lasting immunity to herbivores and necrotrophic pathogens, which involves RNA-directed DNA demethylation and Argonaute 1–mediated opening of chromatin of JA defense genes (Wilkinson et al. 2023). Or perhaps, ARR priming involves the alleviation of transcriptional repression by the AIPP3-CPL2-PHD complex (AIPP3:ASI1-IMMUNOPRECIPITATED PROTEIN3-CPL2:CARBOXYL-TERMINAL DOMAIN PHOSPHATASE-LIKE2-PHD FINGER-CONTAINING PROTEIN2), as has been observed for *Flowering Locus T* (*FT*) (Zhang et al. 2020) and *FMO1* genes (Lan et al. 2023). *FT* and *FMO1* genes were trimethylated at Histone 3 Lysine 27 (H3K27), and these H3K27 methylated sites were recognized by the AIPP3-CPL2-PHD complex, resulting in disruption of transcription of *FT* and *FMO1* (Zhang et al. 2020; Lan et al. 2023). Given that the floral transition can be separated from ARR in short- and long-day conditions and in early- and late-flowering mutants (Wilson et al. 2017), alleviation of repression of *FT* to allow flowering is probably not involved in initiating ARR. Despite the fact that the AIPP3-CPL2-PHD2 gene was not upregulated in the ARR transcriptomes (Tables S2 and S3) or ARR-SAR comparison (Table S2), it is possible that the *FMO1* gene becomes demethylated as plants age, resulting in alleviation of AIPP3-CPL2-PHD2 transcriptional repression, leading to modest expression of *FMO1*, resulting in NHP accumulation between 5 and 6 wpg to initiate the ARR-primed state in mature plants.

### Comparison of ARR, ETI, and PTI transcriptomes

PTI-associated gene expression in young plants responding to virulent *Pst* was absent or low compared to mature ARR-competent plants at both 0.25 and 12 hpi. This was expected, as virulent *Pst* secretes effectors via the T3SS to suppress the PTI response early during infection to promote bacterial multiplication and young plant susceptibility (Xin et al. 2018). For example, Arabidopsis plants inoculated with a low dose of virulent *Pst* (5 × 10^5^ CFU/mL) expressed few defense-associated genes from 1 to 12 hpi, followed by a slow steady increase from 13 to 24 hpi (Mine et al. 2018). Additionally, Arabidopsis inoculated with a high dose of virulent *Pst* (7.5 × 10^7^ CFU/mL) expressed few PTI-associated genes from 6 to 17 hpi compared with high defense-associated gene expression in response to a T3SS mutant that cannot secrete effectors to suppress plant defense, resulting in PTI-associated gene expression and a successful PTI response (Lewis et al. 2015).

Fewer differentially expressed genes were observed during ARR at 12 hpi (472 genes) than at 24 hpi (1,534) and also in comparison to PTI (1,874 genes) and ETI (1,751 genes) at 12 hpi, suggesting that ARR is a slower response than PTI and ETI. However, mature untreated ARR-competent plants displayed elevated expression of *ALD1*, *FMO1*, *RLP23*, and *RPL28*, suggesting these plants are already primed, as has been observed in the systemic leaves of SAR-induced plants (Baum et al. 2019). Additionally, elevated defense-related gene expression in mature mock-inoculated leaves compared to young mock-inoculated leaves at 0.25 and 12 hpi supports the idea that ARR-competent plants were primed, as they responded rapidly and strongly to both mock and *Pst* inoculations. Therefore, it appears as if fewer genes are differentially expressed in ARR-responding plants compared to PTI- and ETI-responding plants at 12 hpi because defense-related genes were already upregulated in mature ARR-competent untreated or mock-inoculated plants at 0.25 hpi.

Additional studies examined differential gene expression during PTI and reported more differentially expressed genes than the PTI RNA-seq study presented here. Bjornson et al. (2021) observed a total of ∼8,000 differentially expressed genes in Arabidopsis in response to flg22 across 6 time points, whereas we reported differentially expressed genes at single time points. The number of significantly upregulated genes observed in this study at 24 hpi with flg22 (1,362 genes) is comparable to the ∼1,500 upregulated genes observed in *Nicotiana benthamiana* at 24 hpi with a PTI-inducing virus (Necira et al. 2024) and the ∼1,250 upregulated genes observed in leaves at 24 h after PTI induction with flg22 in Arabidopsis (Winkelmüller et al. 2021).

### ARR and SAR manifestation transcriptome comparisons

Considerable overlap was observed in the ARR and SAR manifestation to *Pst* stage transcriptomes (766/1,291, 59%; Fig. S9), providing additional support that ARR is similar to SAR. The ARR-SAR comparison revealed genes that were not observed in the ARR transcriptome analysis in Fig. 2. For example, the key SAR regulators SARD1 and CPB60g, as well as 8 additional cysteine-rich receptor kinases (CRK6, 8, 14, 15, 16, 18, 23, 40), were observed in the ARR and SAR manifestation transcriptomes. Comparing the ARR transcriptomes in Tables S3 and S4 to ARR-SAR (Table S2), CRKs 36, 38, and 39 were present in all 3 (Table S4). A number of these cell surface–localized CRKs are thought to be involved in plant immunity. For example, CRK6 and CRK36 interact with the FLS2 flg22 receptor (Yeh et al. 2015; Lee et al. 2017), suggesting they play a role in perceiving bacterial pathogens during PTI. Many CRK genes are upregulated in the ARR transcriptomes and ARR-SAR manifestation comparisons (Table S4), suggesting that CRK upregulation may enhance pathogen perception and/or signaling during both ARR and SAR.

### NHP signaling during ARR

Expression of NHP biosynthesis genes and NHP accumulation in healthy leaves of mature plants was associated with ARR competence, and functional NHP biosynthesis genes were required for ARR, suggesting that NHP signaling is important in establishing an immune ready state in the healthy leaves of mature plants and for the ARR response to *Pst*. In young plants, NHP signaling is required for SAR (Cecchini et al. 2015; Hartmann et al. 2018) and contributes to the transcriptional reprogramming that occurs during the priming of systemic leaves (Bernsdorff et al. 2016; Yildiz et al. 2021), which potentially contributes to the long-lasting and robust resistance observed in SAR-primed leaves. It is possible that NHP signaling in healthy leaves of mature ARR-competent plants contributes to a similar transcriptional reprogramming event, and this idea is supported by the expression of cell surface receptors *RLP23* and *RLP28* in healthy mature ARR-competent plants.

The NHP biosynthesis pathway results in the production of 2 potential signaling molecules: NHP, which requires functional ALD1 and FMO1 to be produced (Ding et al. 2016; Hartmann et al. 2018), and pipecolic acid, which requires only ALD1 (Hartmann et al. 2017). Expression data from mock and *Pst*-inoculated leaves of young and mature plants revealed a rapid upregulation of *ALD1* and *FMO1* at 6 and 12 hpi in ARR-responding plants, suggesting that the NHP biosynthesis pathway is involved in ARR. The importance of ALD1 and FMO1 was confirmed by the ARR defect observed in *ald1-T2* and *fmo1-1* mutants, suggesting that both ALD1 and FMO1 contribute to ARR. Since both ALD1 and FMO1 are required for the biosynthesis of NHP, but FMO1 does not contribute to pipecolic acid synthesis, NHP appears to be more important for ARR than pipecolic acid.

A critical component of ARR is the accumulation of SA in the intercellular space, which requires the expression of SA biosynthesis genes and intracellular accumulation of SA (Cameron and Zaton 2004; Carviel et al. 2014; Wilson et al. 2017). We demonstrated that intracellular and intercellular SA accumulation in response to *Pst* during ARR was dependent on functional NHP biosynthesis genes. A similar phenomenon is observed during SAR, where the accumulation of SA in local leaves is reduced in NHP-deficient plant lines (Bernsdorff et al. 2016; Hartmann et al. 2018; Zeier 2021; Shields et al. 2022). During SAR, the regulation of NHP and SA biosynthesis is regulated by SAR-DEFICIENT1 (SARD1) and CALMODULIN BINDING PROTEIN 60 g (CBP60 g) which bind to promoter regions of SA biosynthesis and transporter genes *ICS1*, *EDS5*, and *PBS3*, as well as the NHP biosynthesis genes *ALD1* and *FMO1* (Sun et al. 2015), resulting in expression of these genes. The regulation of the accumulation of SA and NHP is tightly intertwined in the leaves of young plants, as demonstrated in experiments in which SA treatment results in expression of NHP biosynthesis genes (Ding et al. 2018) and accumulation of the NHP precursor pipecolic acid (Návarová et al. 2012). Moreover, NHP treatment results in the biosynthesis and accumulation of SA (Yildiz et al. 2021). Our work provides support for a similar regulatory relationship between SA and NHP during ARR in response to *Pst*, as demonstrated by reduced accumulation of intracellular SA and reduced downstream *PR1/2* expression in the NHP biosynthesis mutants. However, distant uninoculated leaves of SAR-primed plants displayed a modest accumulation of SA (1- to 2-fold higher than in untreated leaves) (Návarová et al. 2012; Cai et al. 2021), whereas SA levels were not elevated in mature ARR-competent uninoculated leaves, a difference that warrants further investigation.

### NPR1/4 signaling during ARR

In this study, *npr1-1* was partially defective for ARR, whereas *npr4-4D* was fully ARR competent, perhaps because *npr4-4D* mutants contain a wild-type copy of NPR1 (Ding et al. 2018). The NPR4-4D mutant protein in *npr4-4D* is insensitive to SA; therefore, alleviation of NPR4-negative regulation does not occur in young *npr4-4D* plants (Ding et al. 2018). However, NPR1-mediated signaling still occurred in mature *npr4-4D* plants during ARR, as demonstrated by upregulation of *PR1/2*, suggesting that the transcriptional response during ARR depends more on positive regulation of SA-mediated NPR1 signaling and less on SA-mediated alleviation of NPR4 repression. Moreover, both young *npr1-1* and *npr4-4D* were partially compromised in PTI initiated by flg22 or ETI initiated by *Pst* (*avrRpt2*, *avrRps4*) (Liu et al. 2020), including reduced *PR1/2* expression compared to wild-type Col-0 (Liu et al. 2020), suggesting that SA-mediated release of NPR4 repression of downstream defense genes like *PR1* and *PR2* contributes to ETI and PTI.

Our data demonstrate that *npr1-1* is partially defective for ARR. This contrasts with many studies in which SAR to *Psm* or *Pst* was completely abolished in *npr1-1* (Cao et al. 1994; Fu et al. 2012; Lee et al. 2015; Chen et al. 2017), leading to the idea that ARR but not SAR includes NPR1-independent components. However, during SAR assays in which SAR was induced with virulent *Psm* and distant leaves were challenged with virulent *H. parasitica* Noco2, it was shown that *npr1-1* and *npr4-4D* were partially SAR defective compared to the completely SAR-defective double *npr1-1 npr4-4D* mutant (Liu et al. 2020), suggesting that NPR1-independent components are important for SAR, as has been shown in other studies (Bowling et al. 1997; Rairdan et al. 2001; Uquillas et al. 2004).

It is also possible that the partial SAR response in young *npr1-1* is due to the elevated levels of SA that accumulate in response to *Pst avrRpt2* in *npr1-1* compared to wild-type (Delaney et al. 1995; Liu et al. 2020). This makes it tempting to speculate that loss of NPR1-mediated signaling in *npr1-1* is rescued by elevated SA accumulation in *npr1-1* responding to *Pst*, resulting in a partial ARR defect in mature *npr1-1*. We demonstrated that intercellular SA accumulates during both ARR and PTI and is associated with suppression of *Pst* biofilm-like aggregate formation and *Pst* multiplication (Wilson et al. 2017; Xiao et al. 2024). Perhaps elevated intercellular SA accumulates in mature *npr1-1*, where it suppresses *Pst* biofilm-like aggregate formation and multiplication, resulting in a partial ARR response, and for unknown reasons, this rarely happens in young *npr1-1* plants (Liu et al. 2020). Future experiments should examine intercellular SA accumulation in mature *npr1-1*, *npr4-4D*, and *npr1-1 npr4-4D* during ARR.

Young *npr1-1 npr4-4D* plants are fully SAR defective (Liu et al. 2020), and mature *npr1-1 npr4-4D* plants are fully ARR defective (this work), suggesting that NPR1 and NPR4 proteins are involved in both ARR and SAR. During SAR, evidence indicates that NHP signaling is upstream of NPR1 and NPR4 as infiltration of lower leaves with 1 mM NHP did not rescue the SAR defect in *npr1-1 npr4-4D* mutants (Liu et al. 2020). Our evidence suggests that during ARR, NHP also acts upstream of NPR1-mediated signaling as wild-type NHP biosynthesis gene expression was observed in untreated leaves of mature ARR-defective *npr1-1 npr4-4D* plants.

### ARR-competent plants may not be affected by *Pst* coronatine

Several genes involved in the JA response were upregulated in leaves of young plants responding to *Pst*, including *JAZ3* and *JAZ10*, and the JA defense marker genes *VSP1* and *VSP2*. *Pst* secretes the phytotoxin coronatine, a structural analog of JA-Ile, resulting in initiation of the plant JA pathway to promote stomatal reopening and suppress SA-mediated defense responses (reviewed in Geng et al. 2014). None of these JA response genes were upregulated in mature ARR-responding plants, suggesting that ARR-competent plants somehow suppress *Pst* coronatine production or mature ARR-competent plants are not affected by coronatine. Furthermore, coronatine promotes expression of the SA-inactivating genes *BSMT1* and *UGT74F2* in the leaves of young plants (Zheng et al. 2012; Dean and Delaney 2008). Both *BSMT1* and *UGT74F2* were not upregulated in mature plants during ARR to *Pst*, suggesting that *Pst* inactivation of SA via coronatine does not occur or is not effective in ARR-competent mature plants.

### ARR model

This study provides support for the existence of an ARR immune-ready/primed state that is similar to the primed state observed in distant leaves of plants induced for SAR (Vlot et al. 2021). Both SAR and ARR are dependent on NHP biosynthesis and accumulation, as well as cell surface receptor gene expression. Based on the data presented here and ARR studies from our laboratory (Rusterucci et al. 2005; Wilson et al. 2013, 2017), we propose the following ARR model. Between 5 and 6 weeks of age, a currently unknown developmental event results in the initiation of a primed/immune-ready state in healthy leaves of mature plants that is associated with NHP biosynthesis gene expression (*ALD1*, *FMO1*), NHP accumulation, and signaling, resulting in the expression of cell surface immune receptors. This allows mature Arabidopsis to perceive and successfully respond to normally virulent *Pst* by rapidly expressing NHP biosynthesis genes and many other PTI, ETI, and SAR genes, resulting in the production of NHP and antimicrobial intercellular SA. It is possible that chromatin remodeling (Jaskiewicz et al. 2011) and chromatin opening (Baum et al. 2019) at the promoter regions of SAR priming genes such as *FMO1* are also important for the ARR primed state that results in rapid expression of *FMO1* and other SAR/ARR genes in response to *Pst*. This leads us to hypothesize that ARR includes a developmentally controlled immune-ready/primed state similar to the primed state in distant leaves during SAR (Baum et al. 2019) or JA-induced molecular memory in response to herbivory (Wilkinson et al. 2023).

Comparative transcriptomics revealed that ARR shares defense gene expression profiles with PTI, ETI, and SAR, and mutant/gene expression analysis demonstrated a requirement for key PTI, ETI, and SAR genes (*ALD1*, *FMO1*, *NPR1*, *NPR4*) during ARR, providing further evidence that plant immune pathways are not separate distinct pathways but are intertwined and interconnected (Ngou et al. 2021; Yuan et al. 2021).

## Materials and methods

### Plant lines and growth conditions

All plant lines used in this study were in the Columbia (Col-0) background. Col-0, *ald1-T2* (CS65521), *sard4-7* (SALK_053625C), *fmo1-1 (*SALK_026163C), *bkk1-1* (CS71778), and *bak1-5 bkk1-1* (CS71787) seeds were obtained from the Arabidopsis Biological Resource Center, Ohio State University. *bak1-5* was supplied by Dr. J. Monaghan (Queen's University, Kingston, Ontario, Canada). *npr1-1*, *npr4-4D*, and *npr1-1 npr4-4D* seeds were supplied by Dr. Y. Zhang (University of British Columbia, Vancouver, British Columbia, Canada). Seeds were surface-sterilized and stratified in darkness at 4 °C for 2 days. Sterile seeds were sown on Murashige and Skoog medium and placed under continuous light (∼90 μmol m^−2^ s^−1^, 4,100 K fluorescent bulbs) for 5 to 6 days, at which time seedlings were transplanted into soil (Sungro Sunshine mix #1) hydrated with 1 g l^−1^ 20-20-20 fertilizer. Plants were grown under fluorescent (110 ± 1 μmol m^−2^ s^−1^, 4,100 K fluorescent bulbs) in short days (9-h days/15-h dark nights) at 22 ± 2 °C with 80% ± 5% relative humidity. Plants were watered when needed and fertilized every other week with 1 g per liter of 20-20-20 fertilizer for the duration of the experiment.

### *In planta* bacterial growth quantitation

*P. syringae* pv. *tomato* strain DC3000 carrying the empty vector plasmid pVSP61 (*Pst*) was used in all experiments (Whalen et al. 1991). Bacteria were grown to exponential phase in King's B medium [20 g l^−1^ proteose peptone #3, 1% (v/v) glycerol, 1.5 g l^−1^ K_2_HPO_4_] (King et al. 1954) with kanamycin (50 μg mL^−1^). Cells were collected by centrifugation and resuspended in 10 mM MgCl_2_ to 10^6^ CFU mL^−1^, and leaves were inoculated by pressure infiltration with a needleless syringe. For quantification of *in planta* bacterial levels, 3 replicates, each containing 8 leaf discs, were cut from inoculated tissue using a 0.6-mm cork borer. Leaf discs were shaken at 200 rpm for 1 h in 10 mM MgCl_2_ with 0.1% (v/v) Silwet L-77. Serial dilutions were plated on King's B agar plates with kanamycin (50 μg mL^−1^) and rifampicin (100 μg mL^−1^), and CFUs were quantified 2 days later.

### Transcriptomes and analysis

#### Plant material for ARR transcriptomes

Adult leaves (leaf 8 and above) of ARR-incompetent young (3.25 wpg) and ARR-competent mature (6.25 wpg) plants were harvested at 0.25, 12, and 24 hpi with a mock solution (10 mM MgCl_2_) or with 10^6^ CFU/mL *Pst* (hpi). Leaves were immediately flash-frozen in liquid nitrogen and stored at −80 °C. Three biological replicates were collected for each treatment group. Each biological replicate consisted of ∼100 to 250 mg of leaf tissue collected from 9 plants.

#### Plant material for PTI transcriptomes

Adult leaves (leaf 8 and above) of young 3.5 wpg Col-0 plants were infiltrated with 1 µM flg22 or sterile water (mock treatment). At 12 and 24 h postinfiltration, leaves were harvested, flash-frozen in liquid nitrogen, and stored at −80 °C. Three biological replicates were collected for each treatment group. Each biological replicate consisted of ∼100 mg of leaf tissue collected from 3 plants.

#### RNA isolation for sequencing

Frozen leaf tissue (∼100 mg) was ground in a precooled mortar filled with liquid nitrogen. Total RNA was isolated using Invitrogen Trizol reagent according to the manufacturer's instructions. RNA was purified on-column using the Qiagen RNeasy mini kit. Residual DNA was degraded using Sigma-Aldrich DNase I prior to sequencing. The approximate quantity of RNA and presence of organic contaminants were determined using a Thermo Scientific NanoDrop 1000.

#### Library preparation and sequencing

Total RNA samples were purified using oligo-dT magnetic beads to enrich for polyadenylated mRNA transcripts. Enriched mRNA transcripts were reverse transcribed to produce cDNA. cDNAs were fragmented and ligated with adapters. Total RNA in libraries was quantified by electrophoresis using an Agilent TapeStation, then diluted to 10 nM RNA. Samples were sequenced on an Illumina HiSeq flow cell. Library preparation and RNA sequencing were performed by GeneWiz South Plainfield (http://www.genewiz.com).

#### Read and transcript processing of ARR and PTI transcriptomes

Following sequencing, adapter sequences and low sequence-quality base pairs were removed from reads using Trimmomatic v0.36 (Bolger et al. 2014). After trimming reads, forward and reverse read pairs in which both reads were >35 bp were mapped to the Arabidopsis genome (TAIR10) using STAR v2.6.1 (Lamesch et al. 2012; Dobin et al. 2013). For each library, aligned reads were counted per gene using HTSeq v0.11.2 (Anders et al. 2015). Read counts were transferred into the R environment for differential expression analysis (Love et al. 2014). Genes with only a few mapped reads (average count of <10 reads per sample) were discarded. To normalize the expression to the amount of RNA sequenced, each library was normalized using size factors calculated by DESeq2 (Love et al. 2014). The transcript abundance values were shifted by a constant of +1 to allow the data to be log_2_-transformed. Differentially expressed genes were determined using the DESeq2 package (Love et al. 2014) at each time point.

### Gene expression analysis

RNA extraction and cDNA synthesis were performed as described previously (Wilson et al. 2017). Briefly, each biological replicate consisted of RNA isolated from ∼100 to 250 mg leaf tissue harvested at the indicated time points. The optical density at 260 nm of RNA samples was measured using a NanoDrop spectrophotometer to equalize nucleotide content in each sample prior to cDNA synthesis. Following cDNA synthesis, RNA samples (∼250 ng/μL) were diluted 2-fold with nuclease-free water. RT-qPCR was performed using a Bio-Rad CFX-96 real-time PCR detection system in 20-μL reactions consisting of 2 μL template, 400 nM of each primer (annealing temperature 60 °C; Table S5), and 1× SYBR Green mastermix (Bio-Rad). Four biological replicates were analyzed for each sample group. Real-time quantitative PCR data were analyzed using a sigmoidal model in R with the “qpcR” package (Ritz and Spiess 2008). The threshold cycle was defined as the cycle number at the maxima of the second derivative on the sigmoidal curve. After RT-qPCR, the C_T_ values of *SECRETORY5A* (*SEC5A*; AT1G76850) and *CULLIN4* (*CUL4*; AT5G46210) were compared across sample groups to validate their use as reference genes. Expression of *CUL4* and *SEC5A* was independent of age, inoculation, and time point (Fig. S10), supporting the use of *CUL4* and *SEC5A* as reference genes. All expression graphs relative to *CUL4* are available in the Supplementary figures. Raw qPCR data can be found at https://github.com/nunngm/ARR_arabidopsis_transcriptomics.

#### Data collection and processing of ETI transcriptomes

To compare ETI transcriptomes to ARR transcriptomes, RNA-seq reads from Mine et al. (2018) were used, in which leaves were inoculated with 10^6^ CFU/mL *Pst avrRpm1* or a mock solution of 10 mM MgCl_2_ and collected at 12 and 24 hpi (Mine et al. 2018). The raw single-end reads were downloaded from the National Center for Biotechnology Information Gene Expression Omnibus database (GSE88798). Raw reads were handled using the same pipeline described above.

#### Multivariate analysis and sample distance

Principal component analysis was performed on the covariance matrix of *Pst*-responsive genes (>2-fold change, adjusted *P* < 0.05) across RNA-seq libraries to explore variation between transcriptomes using the base R “stats” v4.2.2 package. Log_2_-transformed transcript abundance values associated with 18,736 genes were treated as variables, while each of the 36 RNA-seq libraries was treated as an observation.

The base R “stats” v4.2.2 package was used to calculate pairwise Euclidean distances between samples from normalized log_2_-transformed transcript abundance. Sample distances were then hierarchically clustered using the average linkage method. The resulting clustered distance matrix and dendrogram was visualized using the “gplots” v3.1.3 R package.

#### WGCNA analysis

The weighted gene correlation network analysis was completed using the WGCNA package (Langfelder and Horvath 2008) as described previously in Simopoulos et al. (2020). The code for this section was adapted from Simopoulos et al. (2020). Final scripts used in this study can be found at https://github.com/nunngm/ARR_arabidopsis_transcrtipomics.

#### GO enrichment

GO enrichment was performed using the Bioconductor topGO v2.48.0 R package (Alexa and Rahnenfuhrer 2022). Arabidopsis GO terms were downloaded 20 June 2020 from the Arabidopsis Information Resource database (Lamesch et al. 2012). GO terms among indicated genes were compared to all 18,736 genes expressed in the transcriptome libraries with a false discovery rate significance threshold of 0.05.

#### Data collection and analysis of SAR and ARR transcriptomes

To compare SAR transcriptomes to ARR transcriptomes, the “cP” comparison from Baum et al. (2019) was used, in which lower leaves were inoculated with either 3 × 10^8^ CFU/mL *Psm* or a mock solution of 10 mM MgCl_2_, and 72 hpi upper leaves were systemically challenged with tap water. Upper challenged leaves from *Psm*- or mock-induced plants were collected 2.5 h after they were challenged. Baum et al. (2019) performed RNA-seq and subsequently compared the 2 sets of samples; the supplemental dataset provided alongside Baum et al. (2019) was used to perform the comparison in this study. ARR transcriptomes were analyzed as described above. Significantly upregulated genes were defined as a list of genes from each dataset with adjusted *P* < 0.01 and with a >2-fold difference. The 3 gene lists were compared to generate the Venn diagram. Relevant code can be found at https://github.com/nunngm/ARR_arabidopsis_transcrtipomics.

### Collection of IWFs and leaves minus IWFs

Collection of IWFs from Arabidopsis leaves was completed based on the protocol outlined in O’Leary et al. (2014). Briefly, approximately 500 mg of leaf tissue (6 to 8 leaves) per biological replicate was collected at 12, 18, and 24 h postinoculation and vacuum infiltrated with distilled water. Leaves were blotted dry and centrifuged at 600 × *g* for 5 minutes in cut 50-mL syringes fitted to microcentrifuge tubes. Leaves with IWFs removed were then flash-frozen in liquid nitrogen for intracellular SA quantification. IWFs were weighed and subsequently centrifuged at 13,000 × *g* for 5 min. The IWF supernatants were pipetted into new tubes for SA quantification. IWFs and leaves were stored at −80 °C. The IWF pellets were resuspended in 1 mL of 95% ethanol, and the optical density at 664 nm (chlorophyll) and 700 nm (general contamination) was measured to assess contamination of IWFs with cellular contents (Baker et al. 2012).

### SA quantification

*Acinetobacter* sp. ADPWH_lux is a nonpathogenic Gram-negative bacterium that acts as an SA biosensor as it produces luciferase proportional to the amount of SA present in a sample (DeFraia et al. 2008). Briefly, ADPWH_lux was grown with shaking at 37 °C to an OD_600_ of 0.4. For intercellular SA quantification, samples were mixed in a 1:3.5 ratio of IWF to 0.1 M sodium acetate buffer (pH 5.6). A standard curve was prepared with known amounts of SA added to IWFs collected from unstressed *sid2-2* leaves. For intracellular SA quantification, leaves were ground and mixed to 2.5 μL/mg FW with 0.1 M sodium acetate buffer (pH 5.6), followed by incubation at room temperature for 15 min. Leaf samples were centrifuged at 16,000 × *g* for 15 min at 4 °C, and the supernatant was pipetted into a fresh tube. A standard curve was prepared in leaf (minus IWF) extracts from unstressed *sid2-2* leaves. IWFs or leaves minus IWFs were incubated with ADPWH_lux for 1 h at 37 °C in an opaque 96-well plate, and luminescence was measured on a BioTek plate reader at 490 nm to determine SA concentrations.

### Metabolite extraction and NHP quantification

Leaves of young 3.5 wpg and mature 6.5 wpg plants were inoculated with 10^6^ CFU/mL *Pst* or left untreated. At 24 h postinoculation, 6 to 8 leaves were harvested and flash-frozen in liquid nitrogen. Frozen leaves were ground and divided into ∼100-mg aliquots, then weighed. The metabolites were extracted from ground leaves by adding 1 mL 80% (v/v) methanol per 100 mg leaf tissue. An internal standard, 2-hydroxycyclohexane carboxylic acid (2-HCC; Sigma-Aldrich R548480), was added to the extraction solution at a concentration of 1 ng 2-HCC per mg leaf tissue to allow NHP quantification. Ground tissue and extraction solvent were homogenized by vortexing and incubated at 4 °C with shaking (200 rpm) for 30 min. Metabolite extracts were centrifuged for 5 min at 13,000 × *g*. The supernatant was filtered through a 0.45-µm PTFE syringe filter (ChemScience CS-GLPT2545). Filtered extracts were evaporated to dryness under nitrogen gas and resuspended in equal volumes of water prior to LC-MS/MS analysis.

Quantitative analysis was performed on an Agilent 1200 series HPLC system using a Phenomenex Synergi 4-μm Polar-RP (4.6 × 250 mm) column for separation with a flow rate of 1 mL/min at 30 °C. Mobile phase A consisted of 0.1% formic acid in water, and mobile phase B consisted of 0.1% formic acid in acetonitrile. The mobile phase gradient was as follows (percentages indicate percent mobile phase A): 0 min (95%), 2 min (95%), 6 min (50%), 8 min (5%), 8.1 min (95%), and 12 min (95%). The mass spectrometry was performed using a triple quadrupole Sciex 4000 QTRAP in positive ion mode with the following parameters: 50 psi drying gas, 50 psi nebulizing gas, temperature of 600 °C, ion spray voltage of 5,000 V, and an entrance potential of 10 V. For NHP, a mass filter (Q1) of 146 ± 0.5 *m/z* was used to select precursor analytes. Following fragmentation of pure NHP, the 2 most abundant fragments at 100 and 70 *m/z* (Fig. S11) were selected for quantification. Cai et al. (2021) observed a similar fragmentation pattern for NHP using positive ion mass spectrometry. For 2-HCC, a mass filter of 145 ± 0.5 *m/z* was used to select for precursor analytes. Following fragmentation of pure 2-HCC, the 2 most abundant fragments were at 109 and 81 *m/z* and were selected for quantification. The fragmentation (Q2), declustering, and collision exit potentials were optimized for the 2 most abundant fragments of each analyte. The observed retention times with the above parameters of pure compounds on the Phenomex Synergi Polar-RP column were 3.9 min for NHP and 7.4 min for 2-HCC (Fig. S11).

To quantify NHP, the peak area of the NHP fragment at 100 *m/z* was standardized to the amount of leaf tissue by comparing it to the peak area of the 109 *m/z* 2-HCC fragment, which had a known concentration of 1 ng/mg leaf tissue. The peak area ratio was then compared to peak area ratios generated from a standard curve of varying NHP concentrations with constant 2-HCC concentrations using the 100 *m/z* NHP fragment and 109 *m/z* 2-HCC fragment, respectively. Similar results were observed when using the peak area ratios generated using the 70 *m/z* NHP fragment and 81 *m/z* 2-HCC fragment (Fig. S11). The ratio of the 100 *m/z* NHP fragment to the 70 *m/z* NHP fragment peak area was consistent between pure NHP and plant-produced NHP (Fig. S11).

## Supplementary Material

kiag302_Supplementary_Data

## Data Availability

Raw FASTQ sequencing data are available in a SRA/GEO project. Sequence data from this article can be found in the SRA data libraries under accession numbers PRJNA1462389 (ARR dataset) and PRJNA1462626 (PTI dataset). The code used for statistical analyses, along with all nonsequencing raw data, can be found at https://github.com/nunngm/ARR_arabidopsis_transcriptomics.
