# Supplementary material for: Systemic acquired resistance signaling molecule N-hydroxypipecolic acid is involved in Age-related resistance in Arabidopsis thaliana
Source: Plant Physiol. 2026 May 23;202(1):kiag302. doi: 10.1093/plphys/kiag302 (PMC13579328; doi:10.1093/plphys/kiag302)
Supplement: Supplementary file 1 [file kiag302_supplementary_data.zip › ARR-SAR paper-Supp Figures-May2026-final version.pdf]

Supplementary figures

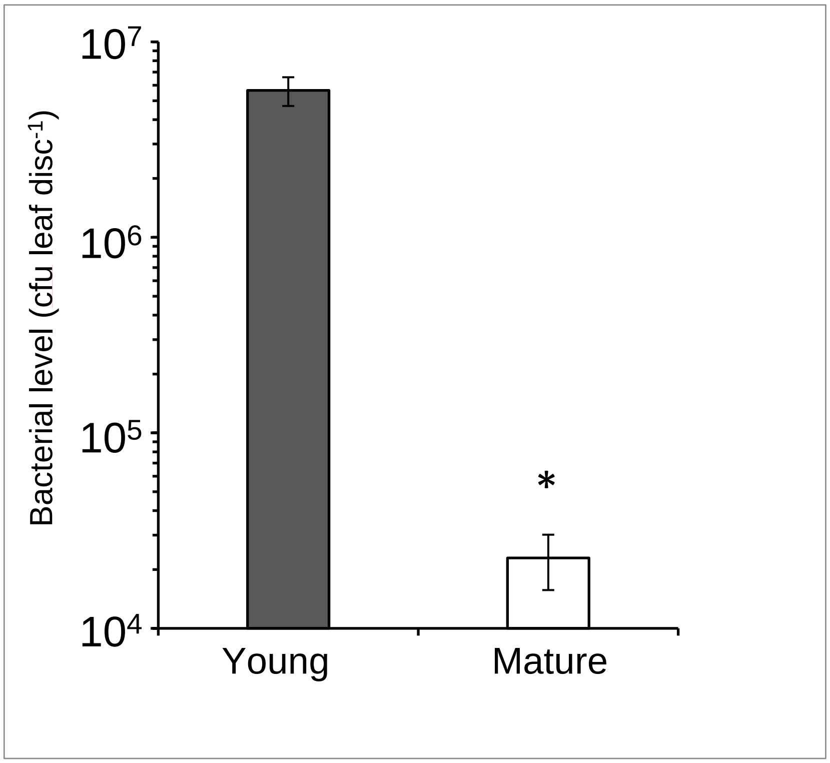

Supplementary Figure S1. Bacterial levels in young and mature plants used for RNA-sequencing. Plants were inoculated with 10^6 cfu/ml *Pst* at either 3.25 or 6.25 wpg and bacterial levels were quantified at 72 hpi. Each bar represents the mean of 3 biological replicates and error bars show standard deviation (Student's T-test, p-value < 0.05).

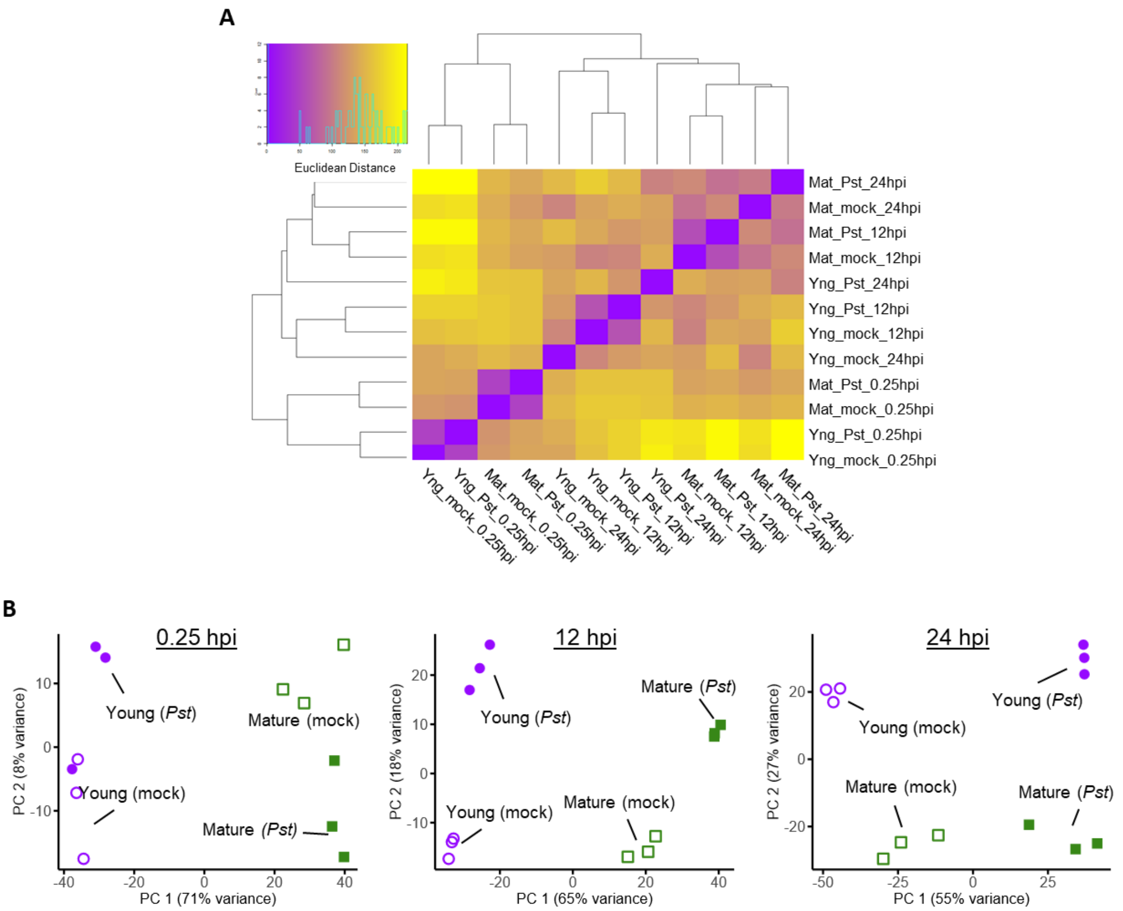

Supplementary Figure S2. Sample distribution of transcriptomes from leaves of young and mature plants after *Pst* inoculation. Young (Yng) and mature (Mat) leaves were inoculated with 10^6 cfu/ml *Pst* or mock-inoculated (10mM $MgCl_2$) and collected at 0.25, 12, and 24 hpi. RNA was extracted and sent for sequencing (A) Heatmap of Euclidean distance between sample groups. Normalised log_2 transcript abundance was used to calculate Euclidean distances between sample groups and cluster samples based on average linkage between clusters. Purple represents smaller distances between samples while yellow represents larger

distances. (B) Principal component analysis biplot of principal component 1 and 2 using transcriptional abundance in leaves of young and mature plants after *Pst* inoculation. Green squares represent transcriptomes from leaves of mature plants and purple circles represent transcriptomes from leaves of young plants. Mock-inoculated samples: open shapes, *Pst*-inoculated samples: closed shapes.

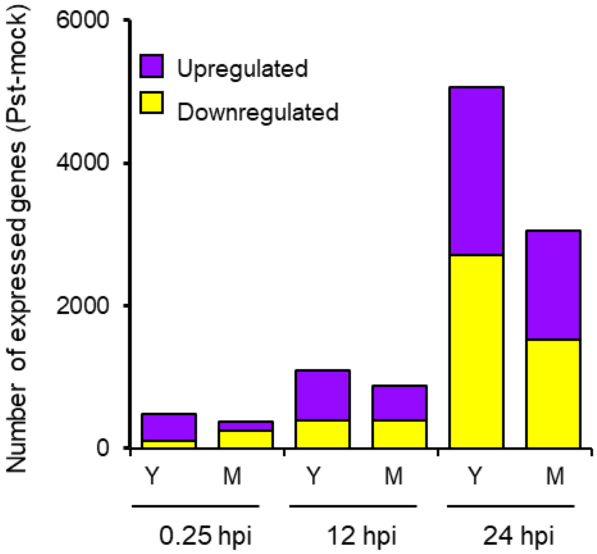

Supplementary Figure S3. The total number of genes responding to *Pst* inoculation in leaves of young (Y) and mature (M) plants over time. Differentially expressed genes were determined by comparing gene expression of mock- and *Pst*-inoculated leaves. Significance was defined as >2-fold change in expression with an adjusted p-value <0.05.

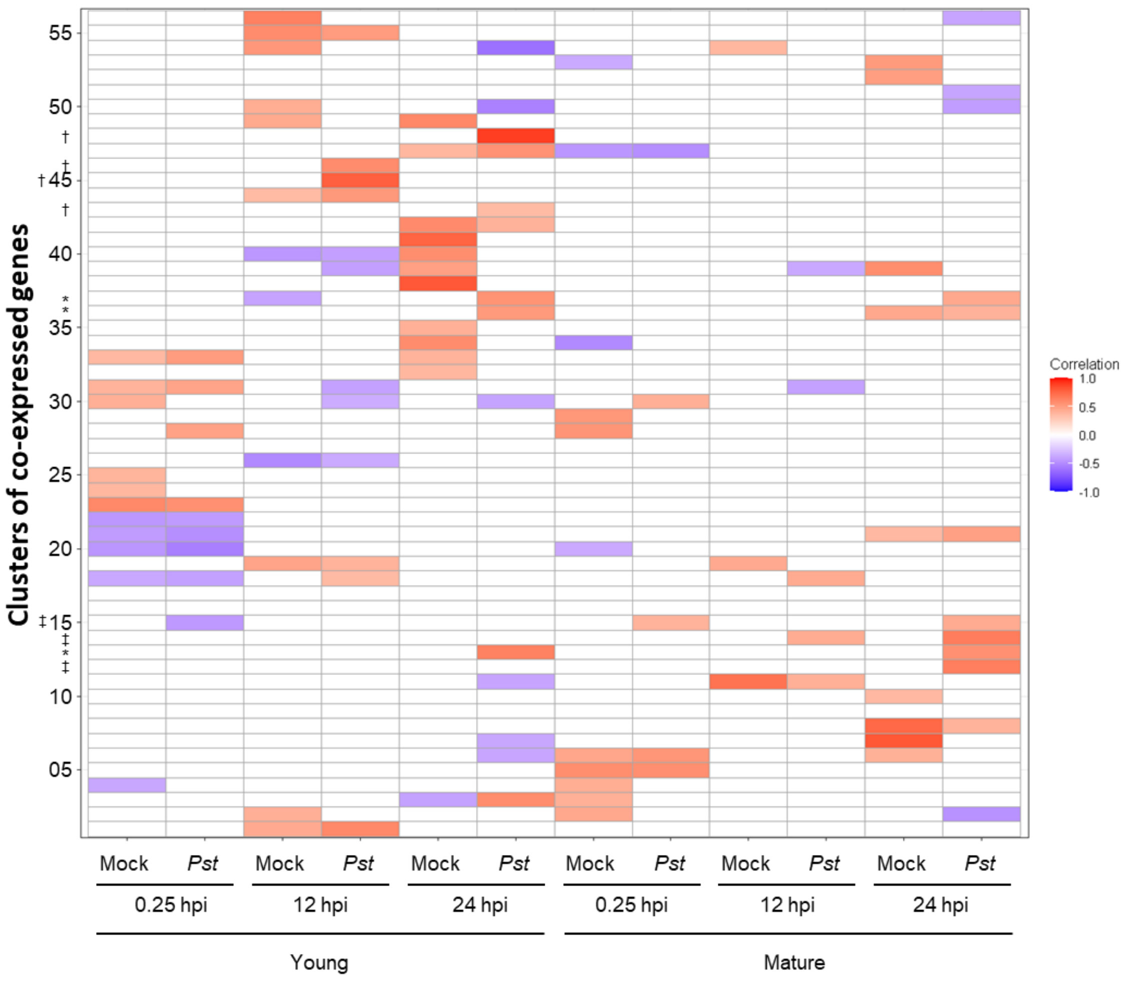

Supplementary Figure S4. Summarised WGCNA of co-expressed gene clusters. Red represents a positive correlation in expression of genes in the indicated cluster. Blue indicates a negative correlation in expression of genes in the indicated cluster. White indicates no statistical significance. Clusters were sorted by expression pattern using hierarchical clustering (Langfelder and Horvath, 2008). * represents clusters of common *Pst*-responsive genes.† represents

clusters expressed in leaves of young plants in response to *Pst*. ‡ represents clusters expressed in leaves of mature plants in response to *Pst*.

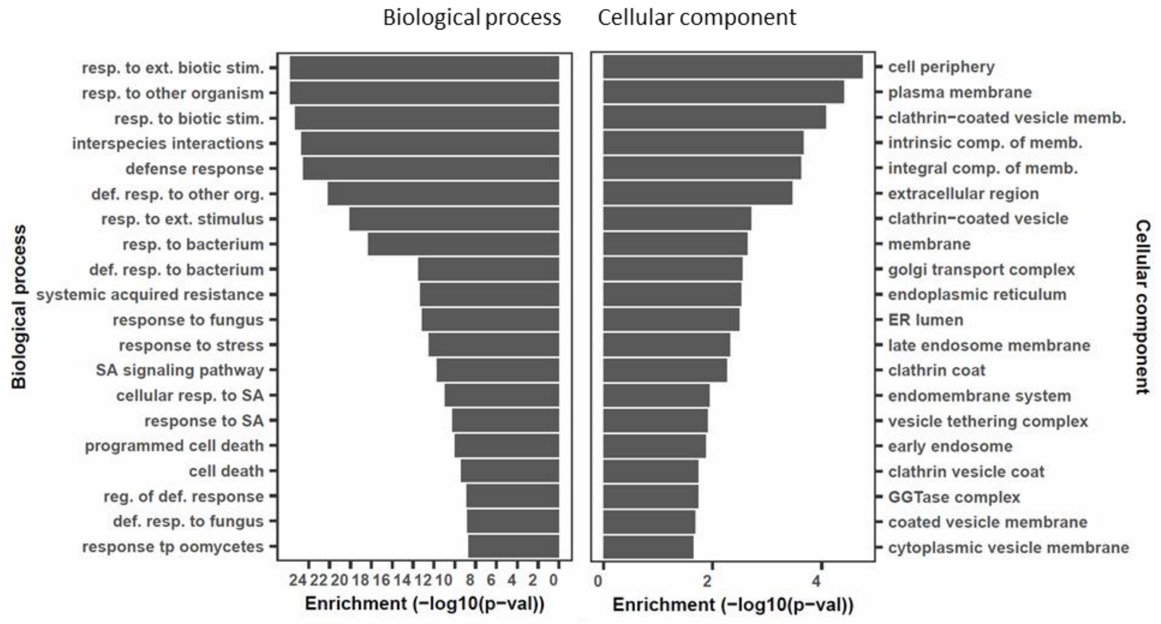

Supplementary Figure S5. Gene ontology (GO) analysis of genes in cluster 14. GO terms associated with genes in cluster 14 from Figure S4 were compared to GO terms found in all expressed genes. The 20 most significantly enriched GO terms among genes in the cluster 14 are displayed.

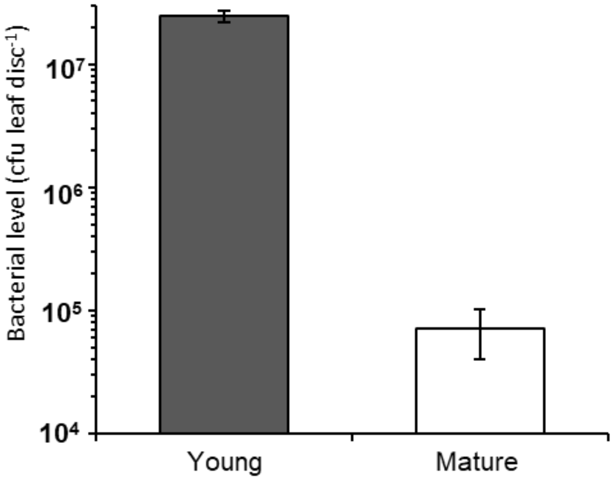

Supplementary Figure S6. Bacterial levels of young and mature plants for Figure 4. Plants were inoculated with 10^6 cfu/ml of *Pst* at either 3.25 or 6.25 wpg and bacterial levels were quantified at 72 hpi. Each bar represents the mean of 3 biological replicates and error bars show standard deviation.

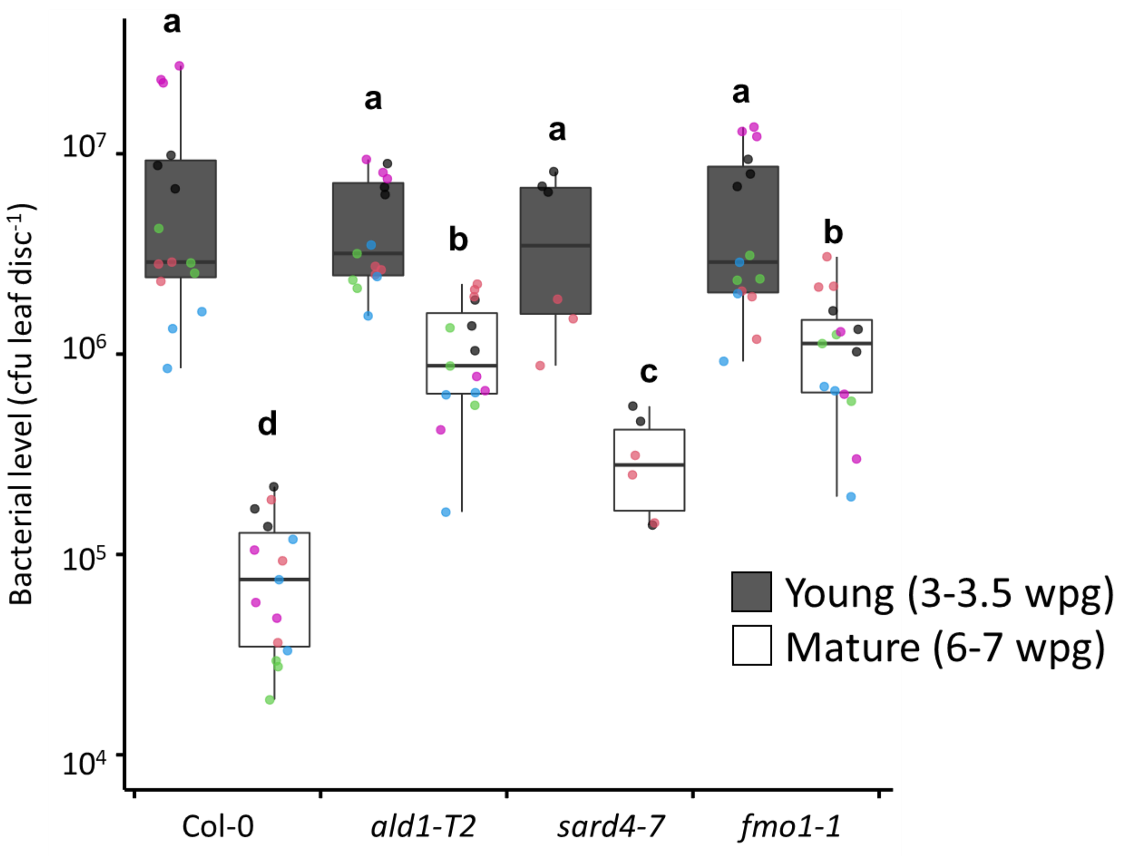

Supplementary Figure S7. NHP biosynthesis gene function is required for ARR. Young (3-3.5 wpg) or mature (6-7 wpg) leaves were inoculated with 10^6 cfu/ml of *Pst* and bacterial levels were quantified at 72 hpi in 5 separate experiments. Each experiment is indicated by different coloured data points that represent 3 biological replicates per experiment (pink, orange, green, blue, black). A boxplot summarizes the data with the median as the center line within the box, the box limits representing quartile 1 (lower) and quartile 3 (upper) that define the interquartile range (IQR), whiskers extending to the most extreme values within 1.5×IQR from the quartiles, and individual points beyond these whiskers indicating

potential outliers. Different letters indicate statistically significant differences (ANOVA, Tukey’s HSD, p-value < 0.05).

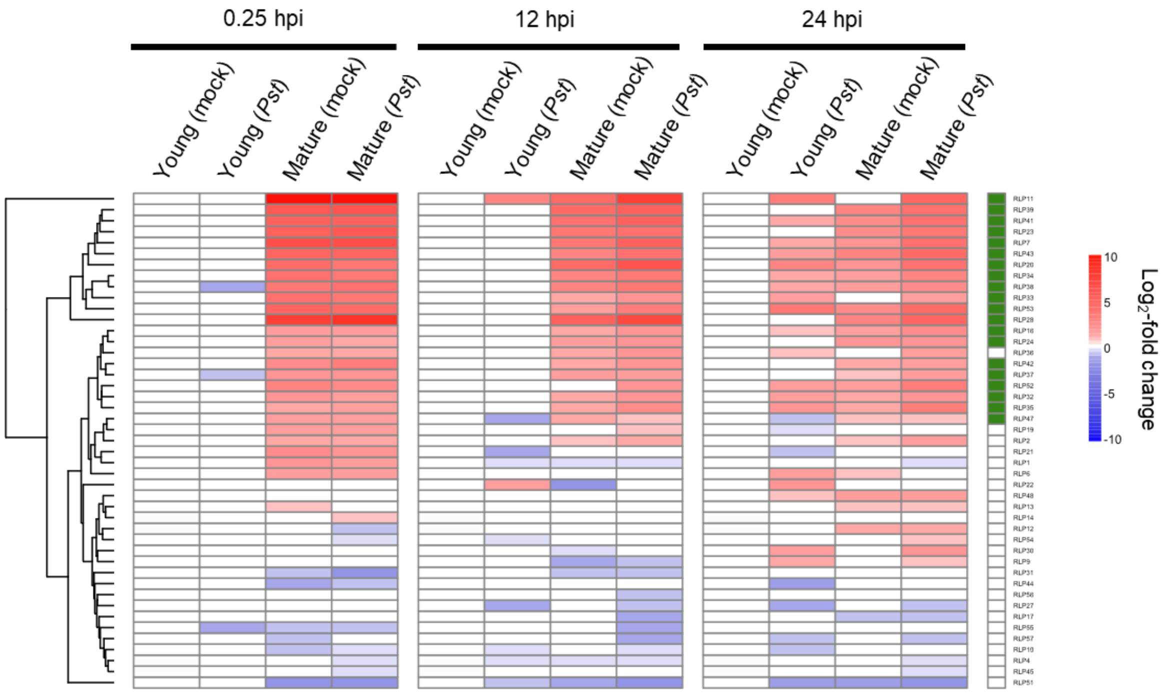

Supplementary Figure S8. Expression heatmap of the RLP gene family following inoculation with *Pst* in the leaves of young and mature plants. Colours represent mean $\log_2$-fold expression of three biological replicates relative to expression in the mock-inoculated leaves of young plants. Red represents significantly increased expression, blue represents significantly decreased expression and white indicates no significant difference. The RLPs present in cluster 14 are marked in green on the right. The $\log_2$-fold differences for each gene were used to calculate Euclidean distance between genes which was used cluster genes based on expression pattern.

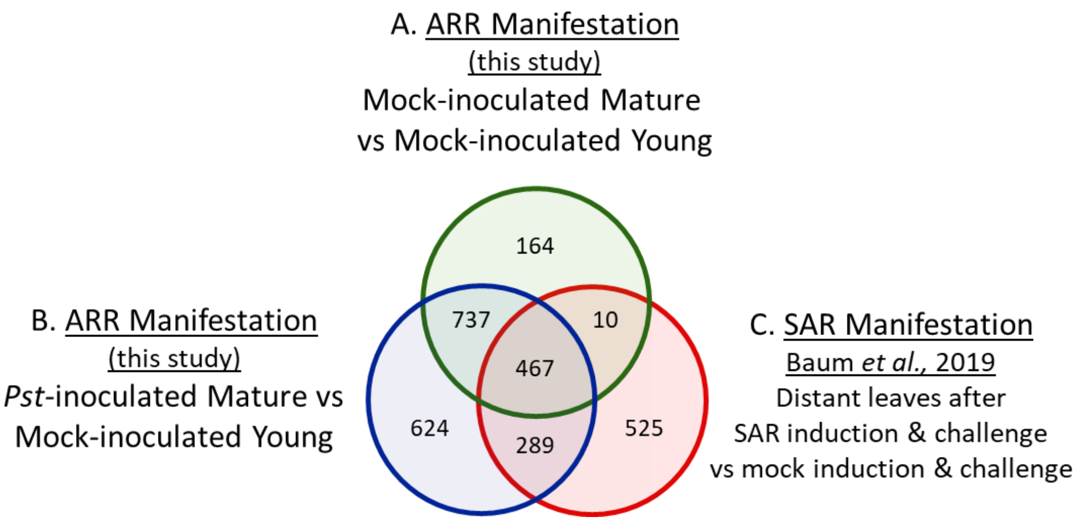

Supplementary Figure S9. Comparison of transcriptional responses during ARR and SAR manifestation. Results (this study) provide evidence that mature plants are primed for ARR, therefore mature plants either mock- **(A)** or *Pst*-inoculated **(B)** represent the ARR manifestation stage and were compared to the SAR manifestation stage (Baum et al., 2019) **(C)**. Significantly upregulated genes in comparisons between A, B and C are displayed in the Venn diagram. **A.** Differential gene expression during ARR manifestation in leaves of mature plants at 12 hours post mock-inoculation compared to mock-inoculated leaves of young plants at 12 hpi. **B.** Differential gene expression during ARR manifestation in leaves of mature plants at 12 hours post *Pst* inoculation (10^6 cfu/ml) compared to mock-inoculated leaves of young plants at 12 hpi. **C.** Differential gene in young plants where local leaves were SAR-induced with *Psm* (3 x 10^8 cfu/ml) and 72 hours later distant leaves were challenge-inoculated with tap water and collected 2.5 hpi during SAR manifestation (Baum et al., 2019), then compared to plants where local

leaves were mock-induced and 72 hours later distant leaves were challenge-inoculated with tap water and collected at 2.5 hpi. The cut-off for differential expression for all datasets was set to a fold-change > 2 and $p_{adj} < 0.01$.

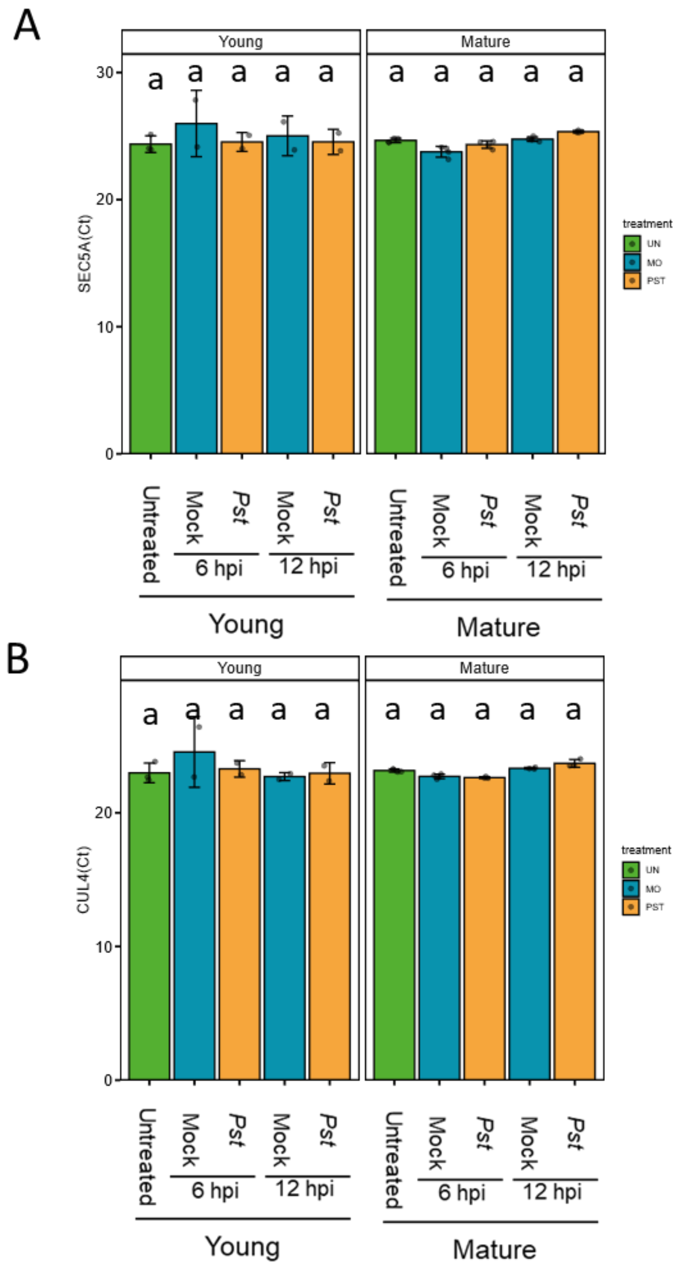

Supplementary Figure S10. Threshold cycle of *SEC5A* and *CUL4* reference genes in young and mature plants. At 3.25 and 6.25 wpg plants were inoculated with 10^6 cfu/ml of *Pst* or a mock solution (10 mM $MgCl_2$). Whole leaves were

collected for RNA extraction from untreated, mock-inoculated or *Pst*-inoculated leaves at the specified timepoints. RNA concentration for each sample was measured on a Thermo-Fisher™ NanoDrop™ ND-1000 Spectrophotometer and subsequently diluted to 250 ng/µl RNA prior to cDNA synthesis. After cDNA synthesis samples were diluted 2x and SsoAdvanced Universal SYBR green master mix was used to perform PCR. qPCR values were measured using a BioRad™ CFX96. Threshold cycles (C_T) were determined as described in methods. Each bar represents the mean of 2 to 4 biological replicates and error bars represent standard deviation. Different letters indicate statistically significant differences (ANOVA, Tukey's HSD, p-value < 0.05). Experiment was performed twice with similar results.

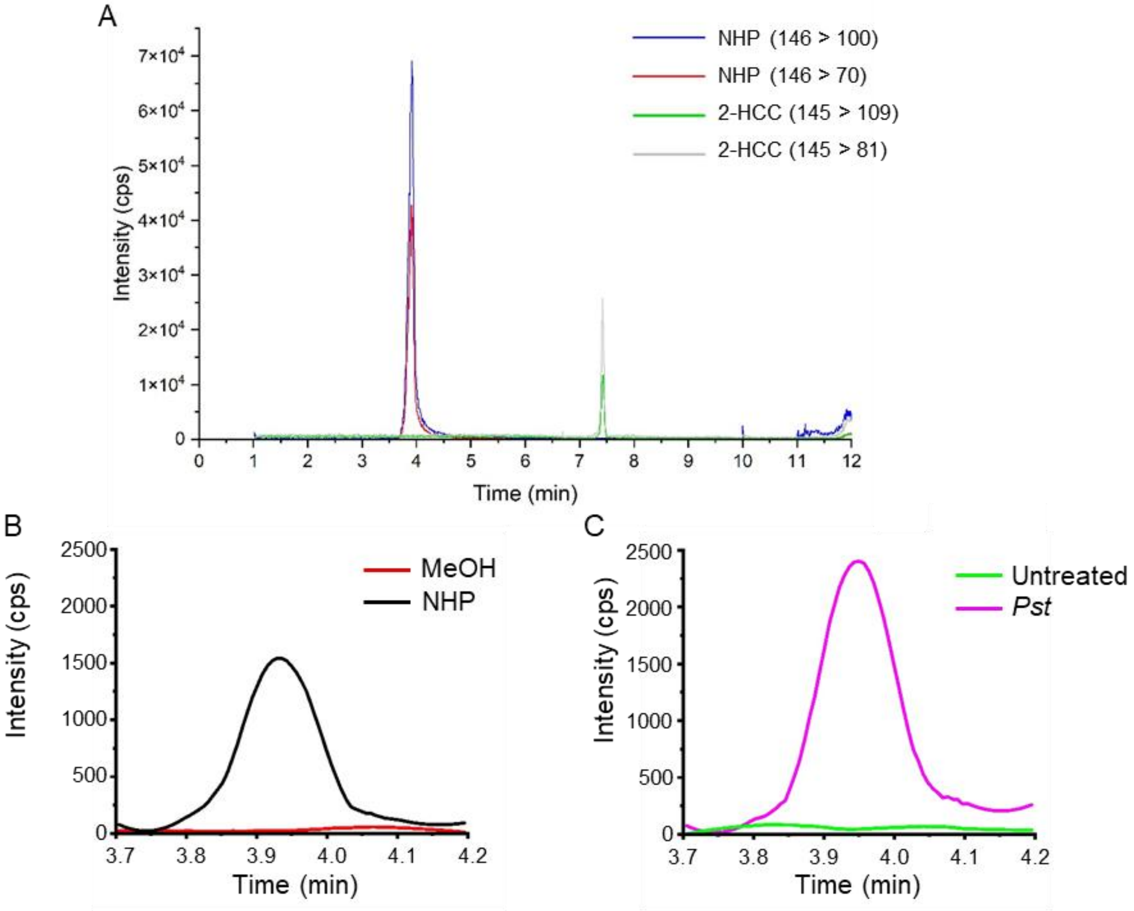

Supplementary Figure S11. LC-MS/MS confirmation of NHP. (A) Multiple reaction monitoring of 250 ng/ml NHP in 80% (v/v) methanol with 50 ng/ml 2-HCC. Brackets display the Q1 > Q3 mass filters for each fragment in m/z ratios. The retention time was 3.9 minutes for NHP and 7.4 minutes for 2-HCC. (B) Metabolites were extracted from untreated leaf tissue of young *fmo1-1* plants. Leaf extracts were split and either spiked with 100 ng/ml NHP or a mock solution (80% methanol). LC-MS/MS was used to detect NHP in each sample (Q1: 146 m/z, Q3:

100 m/z). (C) The leaves of young 3.5 wpg plants were untreated or inoculated with 10^6 cfu/ml of *Pst*. At 24 hpi, leaf tissue was collected for NHP extraction. LC-MS/MS was used to detect NHP in each sample (Q1: 146 m/z, Q3: 100 m/z).

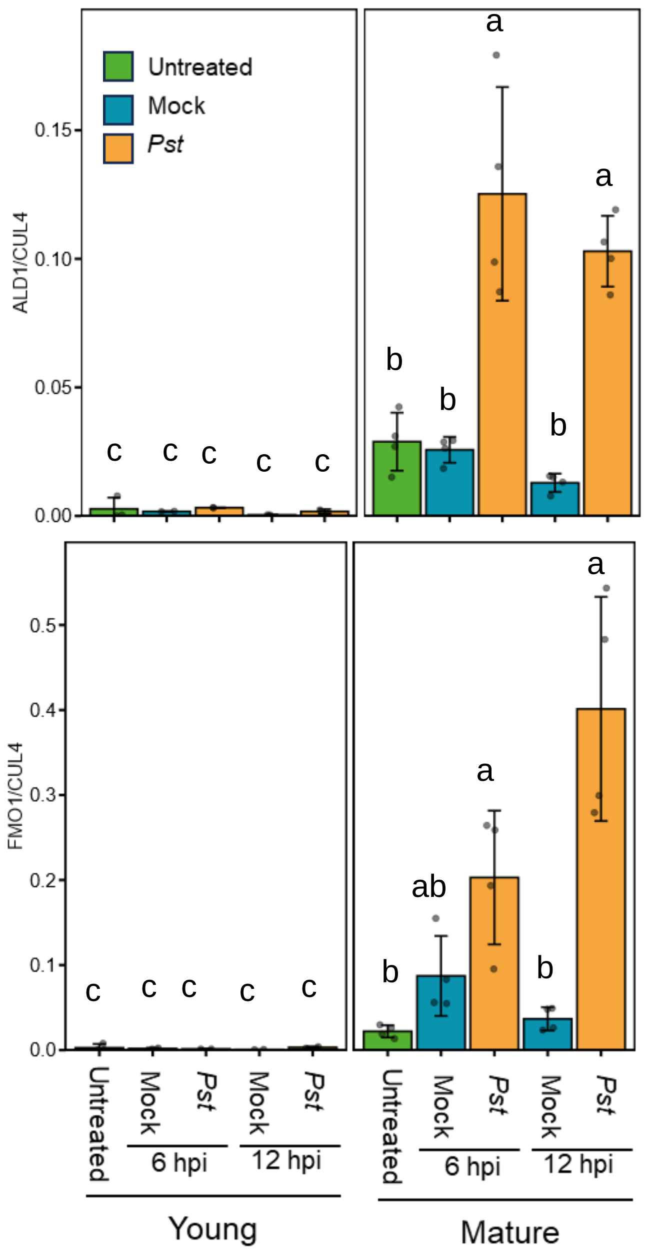

Supplementary Figure S12. *ALD1* and *FMO1* relative to *CUL4* reference gene.

At 3.25 and 6.25 wpg plants were inoculated with 10^6 cfu/ml *Pst* or a mock solution (10 mM $MgCl_2$). At 72 hpi, bacterial levels were quantified (Figure S6). Whole leaves were collected for RNA extraction from untreated, mock-inoculated or *Pst*-

inoculated leaves at the specified timepoints. qPCR was used to examine gene expression of *ALD1* (top) and *FMO1* (bottom) relative to the reference genes *SEC5A* (Figure 4) or *CUL4* (Figure S12). Outliers were calculated using the 1.5 x IQR method and depicted as triangles. Each bar represents the mean of 3 biological replicates and error bars represent standard deviation. Different letters indicate statistically significant differences (ANOVA, Tukey's HSD, p-value < 0.05). Experiment completed twice with similar results.

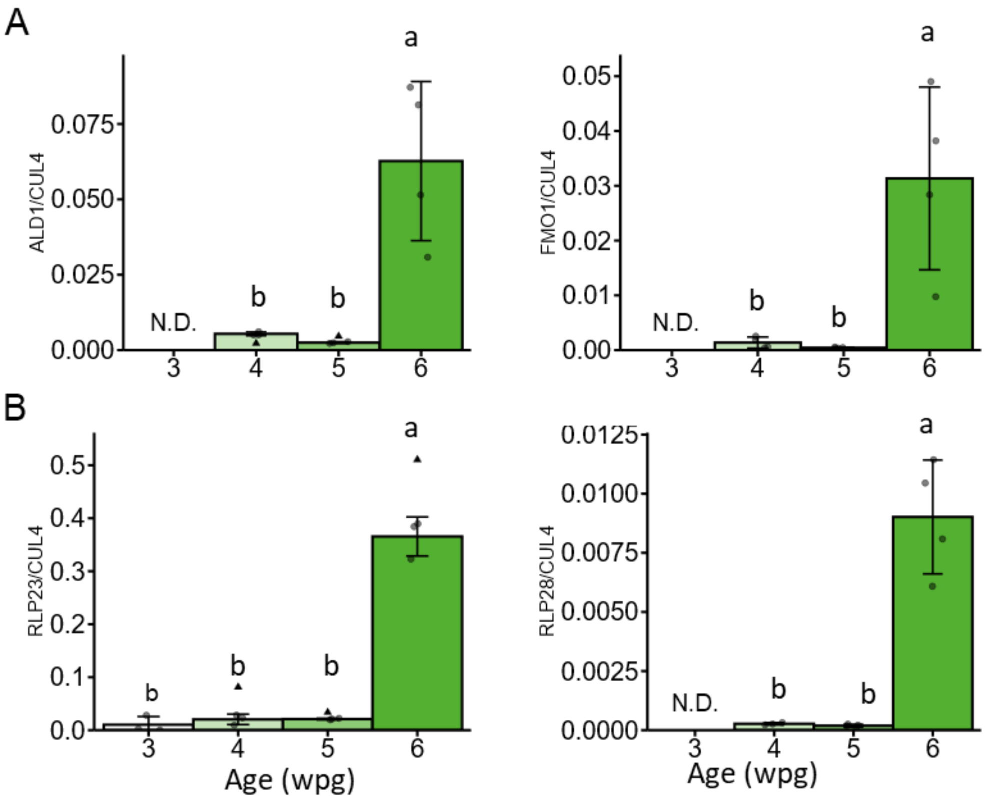

Supplementarty Figure S13. Expression of NHP biosynthesis genes and cell-surface receptors relative to CUL4. Leaves were inoculated each week with 10^6 cfu/ml of *Pst* until the onset of ARR. Bacterial levels were quantified 72 hpi (Figure 6A). Prior to inoculation, leaf tissue was collected for RNA extraction. qPCR was used to examine gene expression relative to the reference genes SEC5A (Figure 8) or CUL4 (above). Outliers were calculated using the 1.5 x IQR method and depicted as triangles. Each bar represents the mean of at least three biological replicates and error bars represent standard deviation. Different letters indicate

statistically significant differences (ANOVA, Tukey's HSD, p-value < 0.05). Expression of ALD1, FMO1, & RLP28 was not detected (N.D.) in 3 wpg samples.

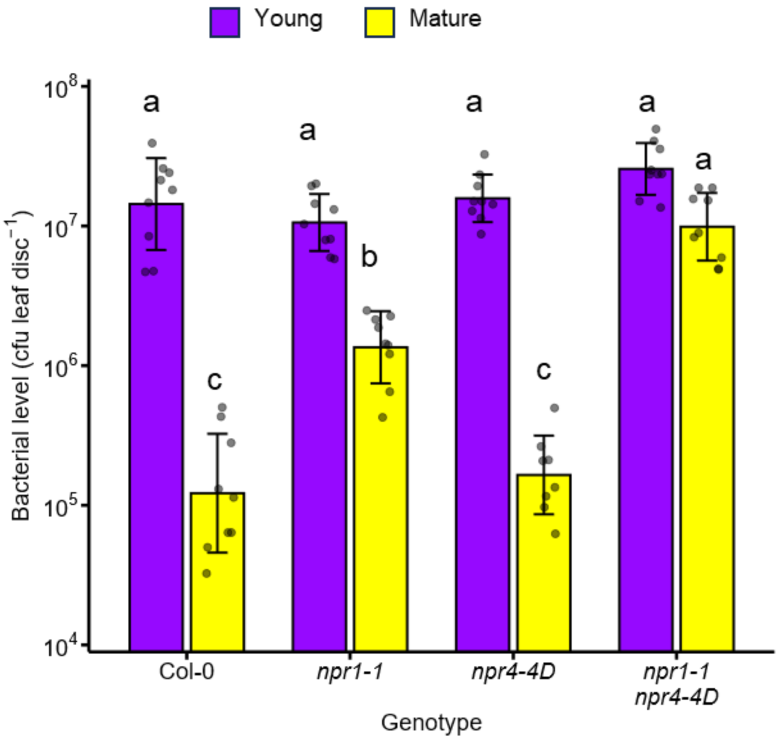

Supplementary Figure S14. Summary of three experiments demonstrating NPR proteins contribute to ARR. Young (3-3.5 wpg) or mature (6-7 wpg) leaves were inoculated with 10^6 cfu/ml of *Pst* and bacterial levels were quantified at 72 hpi. Data points represent colony forming units per leaf disc (n=9). Different letters indicate statistically significant differences (ANOVA, Tukey's HSD, p-value < 0.05).
