# Supplementary material for: Systemic acquired resistance signaling molecule N-hydroxypipecolic acid is involved in Age-related resistance in Arabidopsis thaliana
Source: Plant Physiol. 2026 May 23;202(1):kiag302. doi: 10.1093/plphys/kiag302 (PMC13579328; doi:10.1093/plphys/kiag302)
Supplement: Supplementary file 1 [file kiag302_supplementary_data.zip › Supp Materials-ARR-SAR paper-2025-05-11.docx]

Supplementary Materials

Comparing the magnitude of transcriptional responses to *Pst* in young susceptible and mature ARR-competent plants

The magnitude of the transcriptional response to *Pst* was characterized between young susceptible and mature ARR-competent plants. The transcriptomes of mature and young *Pst*-inoculated leaves were compared to mock-inoculated leaves of their respective ages at each timepoint. DESeq2 analysis was used to identify the number of *Pst*-responsive genes (>2-fold change, adjusted p-value <0.05) in young and mature leaves. At 0.25 hpi, 370 *Pst*-responsive genes were observed in young leaves and 129 in mature leaves (Figure S3). At 12 hpi, ~1000 and ~900 *Pst*-responsive genes were observed in young and mature leaves, respectively (Figure S3). At 24 hpi, the number of *Pst*-responsive genes increased to ~5000 in young and ~3000 in mature leaves (Figure S3). While a similar number of differentially expressed genes were observed in leaves of young and mature plants responding to *Pst*, the clustering of genes expressed in the leaves of mature plants in response to *Pst* (Figure S4) suggests the genes differentially expressed in leaves of mature plants are a separate unique set of resistance-related genes as compared to the genes expressed in leaves of young plants responding to *Pst*.

Susceptible young plant-associated gene expression

Clusters 43, 45, 46 and 48 (†, Figure S4) were expressed only in leaves of young susceptible plants, suggesting these clusters contain genes involved in susceptibility to *Pst*. For example, cluster 48 contained 506 genes expressed specifically in young leaves at 24 hpi. TopGO (Alexa and Rahnenfuhrer, 2022) analysis of this cluster revealed enrichment of genes involved in the jasmonic acid (JA) response, including *VEGETATIVE STORAGE PROTEIN1* (*VSP1*), *VSP2* *JASMONATE ZIM-DOMAIN PROTEIN3* (*JAZ3*), *JAZ10*, and *LIPOXYGENASE2* (*LOX2*). *BENZOATE/SALICYLATE METHYLTRANSFERASE1* (*BSMT1*) and *UDP-GLUCOSYLTRANSFERASE74 F2* (*UGT74F2*), two genes involved in the inactivation of the plant defense hormone salicylic acid through methylation and glucose conjugation, respectively, were also present in this cluster (*BSMT1*: Chen et al., 2003; *UGT74F2*: Dean and Delaney, 2008; Dempsey et al., 2011). This young plant leaf gene expression data is consistent with numerous experiments in which *Pst* was shown to secrete effectors and produce coronatine resulting in suppression of SA-mediated resistance by activating the JA response to facilitate *in planta* multiplication (Geng et al., 2014; Toruño et al., 2016). Given that *Pst* grew to high levels in young Col-0 plants (Figure S1) and presumably was secreting effectors and producing coronatine, it was unsurprising to observe the expression of JA-responsive and SA-inactivating genes. In contrast, mature ARR-responding leaves did not express JA-responsive or SA-inactivating genes and were highly resistant to *Pst,* suggesting that *Pst’s* effectors and coronatine have little effect on mature ARR-competent plants.

Genes associated with susceptibility to *Pst* in young plants

Cluster 48 contained 506 genes expressed specifically in young leaves at 24 hpi. TopGO (Alexa and Rahnenfuhrer, 2022) analysis of this cluster revealed enrichment of genes involved in the jasmonic acid (JA) response, including *VEGETATIVE STORAGE PROTEIN1* (*VSP1*), *VSP2* *JASMONATE ZIM-DOMAIN PROTEIN3* (*JAZ3*), *JAZ10*, and *LIPOXYGENASE2* (*LOX2*). *BENZOATE/SALICYLATE METHYLTRANSFERASE1* (*BSMT1*) and *UDP-GLUCOSYLTRANSFERASE74 F2* (*UGT74F2*), two genes involved in the inactivation of the plant defense hormone salicylic acid by methylation and glucose conjugation, respectively, were also present in this cluster (*BSMT1*: Chen et al., 2003; *UGT74F2*: Dean and Delaney, 2008; Dempsey et al., 2011). This young plant leaf gene expression data is consistent with numerous experiments in which *Pst* was shown to secrete effectors and produce coronatine resulting in suppression of SA-mediated resistance by activating the JA response to facilitate *in planta* multiplication (Geng et al., 2014; Toruño et al., 2016). Given that *Pst* grew to high levels in young Col-0 plants (Figure S1) and presumably was secreting effectors and producing coronatine, it was unsurprising to observe the expression of JA-responsive and SA-inactivating genes. In contrast, mature ARR-responding leaves did not express JA-responsive or SA-inactivating genes and were highly resistant to *Pst* (Figure S1)*,* suggesting that *Pst’s* effectors and coronatine have little effect on mature ARR-competent plants.

Magnitude of transcriptional responses in young and mature plants

The transcriptomes of mature and young *Pst*-inoculated leaves were compared to mock-inoculated leaves of their respective ages at each timepoint to compare the magnitude of the transcriptional respnses. DESeq2 analysis was used to identify the number of *Pst*-responsive genes (>2-fold change, adjusted p-value <0.05) in young and mature leaves. At 0.25 hpi, 370 *Pst*-responsive genes were observed in young leaves and 129 in mature leaves (Figure 3). At 12 hpi, ~1000 and ~900 *Pst*-responsive genes were observed in young and mature leaves, respectively (Figure S3). At 24 hpi, the number of *Pst*-responsive genes increased to ~5000 in young and ~3000 in mature leaves (Figure S3). Similar numbers of differentially expressed genes were observed in leaves of young and mature plants responding to *Pst*, however the clustering of genes expressed in the leaves of mature plants in response to *Pst* (Figure S4) suggests the differentially expressed genes in leaves of mature plants are a separate unique set of resistance-related genes compared to the genes expressed in leaves of young plants responding to *Pst*.

ARR genes not expressed during PTI or ETI

During the Venn diagram analysis (Figure 3), several auxin-related genes were upregulated during ARR, but not during PTI or ETI at 24 hpi, including genes involved in the biosynthesis of auxin, including *IAA-LEUCINE RESISTANT-LIKE GENE5* (*ILL5*), *ILL6*, *NITRILASE2* (*NIT2*), *NIT3*, *AUXIN UPREGULATED3* (*AUR3*) and *UGT74E2*, and auxin-inducible genes like *AUXIN-REGULATED GENE INVOLVED IN ORGAN SIZE* (*ARGOS*), *SOLUBLE EPOXIDE HYDROLASE* (*SEH*), and *ATP-BINDING CASSETTE B4* (*ABCB4*). Auxin-related genes are upregulated in the leaves of young plants in response to virulent *P. syringae* (Wang et al., 2007; Yang et al., 2024) and are thought to enhance susceptibility (Park et al., 2007; Mutka et al., 2013) by suppressing SA-mediated defense responses (Mutka et al., 2013). Therefore, expression of the genes involved in the biosynthesis of auxin were examined in the leaves of both young and mature plants using the ARR dataset*.* While the leaves of mature plants expressed auxin-related genes in response to *Pst*, these genes were more highly expressed in the leaves of young plants responding to *Pst* (ARR RNA-sequencing dataset). For example at 24 hpi, *ILL5* and *UGT74E2* were expressed 28- and 6-fold more in the leaves of young plants compared to the leaves of mature plants inoculated with *Pst* and may explain why ARR-responding plants were highly resistant to *Pst* compared to young plants.
