# Supplementary material for: Systemic acquired resistance signaling molecule N-hydroxypipecolic acid is involved in Age-related resistance in Arabidopsis thaliana
Source: Plant Physiol. 2026 May 23;202(1):kiag302. doi: 10.1093/plphys/kiag302 (PMC13579328; doi:10.1093/plphys/kiag302)
Supplement: Supplementary file 1 [file kiag302_supplementary_data.zip › Table S4. CRKs identified in tables S1 S2 S3.docx]

| Table S5, CRKs identified in Tables S2, S3 and S4 | | | | | |
| --- | --- | --- | --- | --- | --- |
| All CRKs found in Tables S2,3,4 | CRKs in  Table S2  Mature *Pst*-inoculated compared to mature mock-inoculated at 12 hpi | CRKs in  Table S3  Mature *Pst*-inoculated compared to mature mock-inoculated at 24 hpi | CRKs in  Table S4  ARR & SAR manifestation  (see Figure S9 legend for details) | CRKs common to all S2,S3,S4 | CRKs only  in S4  ARR & SAR manifestation  (see Figure S9 legend for details) |
| CRK6 |  |  | CRK6 |  | CRK6 |
| CRK7 | CRK7 |  | CRK7 |  |  |
| CRK8 |  |  | CRK8 |  | CRK8 |
| CRK13 |  | CRK13 |  |  |  |
| CRK14 |  |  | CRK14 |  | CRK14 |
| CRK15 |  |  | CRK15 |  | CRK15 |
| CRK16 |  |  | CRK16 |  | CRK16 |
| CRK18 |  |  | CRK18 |  | CRK18 |
| CRK20 |  | CRK20 |  |  |  |
| CRK23 |  |  | CRK23 |  | CRK23 |
| CRK24 | CRK24 |  | CRK24 |  |  |
| CRK28 | CRK28 |  |  |  |  |
| CRK31 | CRK31 | CRK31 |  |  |  |
| CRK36 | CRK36 | CRK36 | CRK36 | CRK36 |  |
| CRK37 | CRK37 |  | CRK37 |  |  |
| CRK38 | CRK38 | CRK38 | CRK38 | CRK38 |  |
| CRK39 | CRK39 | CRK39 | CRK39 | CRK39 |  |
| CRK40 |  |  | CRK40 |  | CRK40 |
