# Supplementary material for: Systemic acquired resistance signaling molecule N-hydroxypipecolic acid is involved in Age-related resistance in Arabidopsis thaliana
Source: Plant Physiol. 2026 May 23;202(1):kiag302. doi: 10.1093/plphys/kiag302 (PMC13579328; doi:10.1093/plphys/kiag302)
Supplement: Supplementary file 1 [file kiag302_supplementary_data.zip › Table S5. Primers used in this study.docx]

**Table S1. PCR primers used in this study.**

| **Primer** | **Sequence (5’ to 3’)** |
| --- | --- |
| ALD1-F | GTGTTCGGCTTGGTTGGTC |
| ALD1-R | CTCCGTTGAAGGAAGTGGTCA |
| FMO1-F | TCTTCTGCGTGCCGTAGTTTC |
| FMO1-R | CGCCATTTGACAAGAAGCATAGTC |
| RLP23-F | CTGGTATTGTGGCCTGTCGT |
| RLP23-R | CGTCGAGTTATCGCACCAGA |
| RLP28-F | CCCGTTCTTGATCTTGTTGCTT |
| RLP28-R | TCGCACCAGACTCCATTAGAG |
| PR1-F | GCAATGGAGTTTGTGGTCAC |
| PR1-R | GTTCACATAATTCCCACGAGG |
| PR2-F | ATGCTACGGGATGCTAGGCG |
| PR2-R | TCTCCGACACCACGATTTCC |
| SEC5A-F | ACACTCTTGTAGCTGTCCATGC |
| SEC5A-R | TGTCGATTAGACCTTCGATCAGC |
| CUL4-F | AAGGAGCTGGCTGTTTCCC |
| CUL4-R | TGTCCTCTATGCTGGTGGAATC |
